# Targeted disruption of the *cls* gene in *Buchnera aphidicola* impairs membrane integrity and host symbiont dynamics

**DOI:** 10.1016/j.isci.2025.113178

**Published:** 2025-07-22

**Authors:** Kathrine Xin Yee Tan, Shuji Shigenobu

**Affiliations:** 1 Department of Basic Biology, School of Life Science, The Graduate University for Advanced Studies (SOKENDAI), 38 Nishigonaka, Myodaiji, Okazaki, Aichi 444-8585, Japan; 2 Laboratory of Evolutionary Genomics, National Institute for Basic Biology, Okazaki 444-8585, Japan; 3 Life Science Center for Survival Dynamics, Tsukuba Advanced Research Alliance (TARA), University of Tsukuba, Tsukuba 305-8477, Japan

**Keywords:** Natural sciences, Microbiology, Bacteriology, Evolutionary biology, Evolutionary developmental biology, Agricultural science

## Abstract

The obligate symbiosis between pea aphids (*Acyrthosiphon pisum*) and *Buchnera aphidicola* represents metabolic interdependence between the host insect and its bacterial symbiont. *Buchnera* has a highly reduced genome that has lost nearly all phospholipid synthesis genes except *cls*, encoding a cardiolipin synthase homologue. We employed *in vivo* antisense, cell-penetrating peptide (CPP)-conjugated synthetic peptide nucleic acids (PNAs) to knock down *cls* in *Buchnera*. This intervention resulted in significant downregulation of *cls* expression, lowered *Buchnera* titers, pronounced morphological distortions, and reduced aphid reproduction. Notably, *Buchnera* cells were often detected in the aphid gut following anti-*cls* PNAs treatment, deviating from their typical intracellular niche within bacteriocytes. Collectively, the *cls* gene is critical for maintaining *Buchnera* integrity, proper cellular localization, and symbiont-host interactions. Given that the retention of *cls* is a common feature among many obligate endosymbionts despite massive gene loss, our findings offer key insights into the evolutionary principles shaping symbiotic relationships involving membrane biology.

## Highlights

• PNAs were used to investigate the *cls* gene function in an unculturable endosymbiont
• *cls* disruption lowers *Buchnera* titer, alters morphology, and reduces aphid fecundity
• *Buchnera* mislocalized to the gut instead of bacteriocytes following *cls* knockdown
• Conserved *cls* homologs highlight membrane roles in the evolution of endosymbiosis

## Graphical abstract

**Figure:**
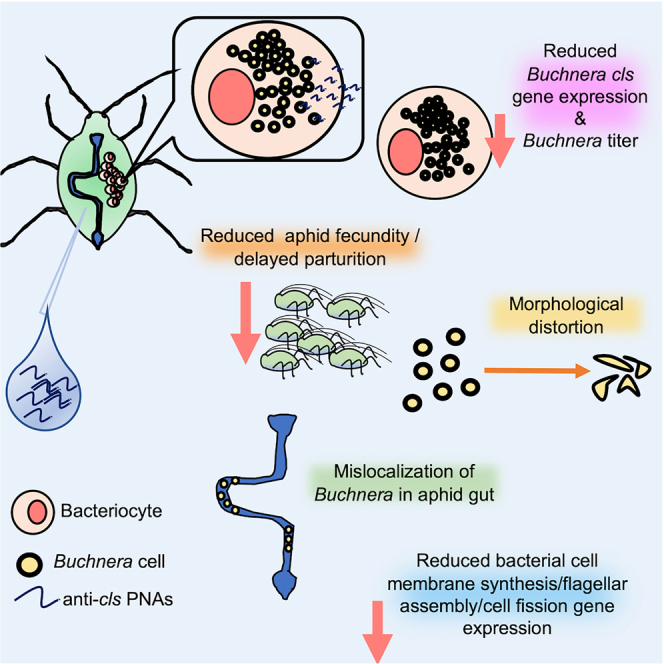

## Introduction

The long-standing association between the pea aphid and *Buchnera aphidicola*, which originated approximately 200 million years ago, serves as a model system for studying endosymbiosis.^1^^,^^2^ This symbiotic relationship has been extensively investigated across various areas, such as genomic evolution, metabolic interdependence and ecological adaptation.^1^^,^^2^^,^^3^^,^^4^ *Buchnera,* a member of the Gamma-Proteobacteria, is an obligate intracellular symbiont of aphids.^1^^,^^3^^,^^4^ It is a Gram-negative bacterium and apparently maintains a typical Gram-negative bacterial membrane consisting of an outer membrane, a peptidoglycan layer, and a cytoplasmic membrane layer, while a Gram-positive bacterial membrane comprises a peptidoglycan layer followed by a cytoplasmic membrane layer.^5^^,^^6^

**Figure 1:**
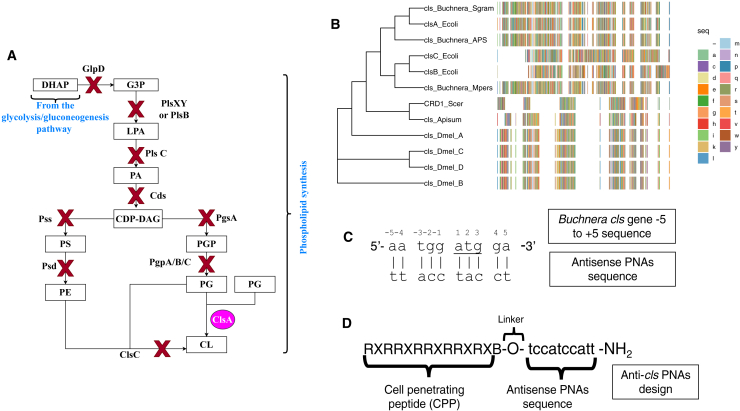
Phospholipid synthesis and design of antisense PNAs targeting *cls* in *Buchnera* (A) The *Buchnera aphidicola* genome retains only one phospholipid synthesis gene, *clsA* (purple circle), while other genes in the pathway are absent, compared to *E*. *coli*. Lipid abbreviations: DHAP: dihydroxyacetone phosphate; G3P: glycerol-3-phosphate; LPA: lysophosphatidic acid; PA: phosphatidic acid; CDP-DAG: cytidine diphosphate-diacylglycerol; PS: phosphatidylserine; PE: phosphatidylethanolamine; PGP: phosphatidylglycerol phosphate; PG: phosphatidylglycerol; CL: cardiolipin. Gene abbreviations: GlpD (G3P dehydrogenase), PlsXY/PlsBC (G3P acyltransferases), Cds (CDP-DAG synthase), Pss (phosphatidylserine synthase), Psd (phosphatidylserine decarboxylase), PgsA (phosphatidylglycerolphosphate synthase), PgpA/B/C (phosphatidylglycerolphosphate phosphatase), ClsA (bacterial cardiolipin synthase), ClsC (alternative cardiolipin synthase). Adapted from Shigenobu et al. (2000), Sohlenkamp & Geiger (2016), and KEGG pathway^6^^,^^8^^,^^9^. (B) Phylogenetic analysis shows *A. pisum*’s *Buchnera* Cls clusters closely with *E. coli* ClsA, while *clsB* and *clsC* are absent. The eukaryotic cardiolipin synthase, CRD1, catalyzes CL synthesis using phosphatidylglycerol (PG) and CDP-DAG. (Dmel = *Drosophila melanogaster*, Scer = *Saccharomyces cerevisiae*, Buchnera_APS = *Buchnera* strain APS, Buchnera_Sgram = *Buchnera* strain *Schizaphis graminum*, Buchnera_Mpers = *Buchnera* strain *Myzus persicae*, Apisum = *Acyrthosiphon pisum*, Ecoli = *Escherichia coli*) (C) The antisense PNAs design targets *Buchnera* genes from −5 to +5 relative to the start codon for interference. The *cls* sequence (−5 to +5) includes the underlined start codon (ATG) and the reverse complementary PNAs sequence conjugated to an arginine-rich cell-penetrating peptide (CPP) (D). See also Tables S1 and S2.

The membrane bilayer is crucial for the integrity and function of bacterial cells, providing a barrier that regulates the passage of molecules in and out of the cell. Phospholipids, the main components of the membrane bilayer, play a vital role in maintaining cell structure and function. Alterations in membrane phospholipid composition are pivotal for bacterial survival and adaptation to environmental stressors.^7^ Intriguingly, *Buchnera* has lost almost all genes for phospholipid synthesis, except for the *clsA* gene.^8^^,^^9^ In comparison to related bacterial species such as *Escherichia coli,* which retains 13 core genes that allow the bacterium to synthesize all major phospholipid classes, such drastic losses of phospholipid synthesis genes in *Buchnera* are an extraordinary feature never observed in free-living bacteria (Figure 1A).^6^^,^^8^^,^^9^ Based on the information from the KEGG database (KEGG data: buc00550; https://www.kegg.jp/pathway/buc00550) and the genome analysis by Shigenobu et al. (2000), *Buchnera* possesses a significant number of genes related to murein synthesis (12 genes involved in peptidoglycan synthesis), yet retains only a single gene related to phospholipid synthesis, namely *clsA* (Figure 1B).^8^^,^^9^

In *E.coli*, the ClsA enzyme condenses two phosphatidylglycerol (PG) to form CL. Additionally, *E.coli* produces CL using the *clsB* gene product, and a third cardiolipin synthase gene, *clsC,* was discovered in 2012.^10^ The ClsC enzyme catalyses the formation of CL with PG and phosphatidylethanolamine (PE) instead of two molecules of PG. Phylogenetic analysis indicates that *Buchnera* possesses a cardiolipin synthase homologous to *E. coli* ClsA, while *clsB* and *clsC* are absent in the *Buchnera* genome.^9^ Moreover, *Buchnera* lacks the ability to produce the three major phospholipids found in *E. coli*, namely CL, PG, and PE, as well as phosphatidylserine (PS), phosphatidylglycerol phosphate (PGP), due to the loss of the necessary biosynthetic genes (Figure 1A). Nevertheless, *Buchnera* has retained the genes involved in glycolysis/gluconeogenesis, enabling it to produce the glycerol phosphate precursor known as glycerone phosphate or dihydroxyacetone phosphate (DHAP).^8^^,^^9^ DHAP can then be converted to sn-glycerol-3-phosphate (G3P), a critical precursor for phospholipid synthesis.

*Buchnera* is unable to convert DHAP to G3P due to the absence of the gene encoding the enzyme, sn-glycerol-3-phosphate dehydrogenase (GlpD).^8^^,^^9^^,^^11^ Despite this limitation, *Buchnera* retains a typical Gram-negative membrane system, which raises an intriguing question of how this symbiotic bacterium maintains membrane integrity without the ability to synthesize key phospholipids.^5^ This apparent paradox underscores the importance of the remaining phospholipid synthesis-related gene, *cls*, in the *Buchnera* genome. Notably, CL and PG are generally essential for the formation of the “Z-ring,” a protein complex required for binary fission, as well as for regulating the formation of the division plane and DNA replication.^12^^,^^13^ Thus, the retention of *cls* in *Buchnera* highlights its central role in maintaining cellular integrity and functionality, despite the loss of other phospholipid biosynthesis pathways.

Previously, we had successfully devised an effective gene manipulation technique for obligate endosymbionts using antisense cell penetrating peptide (CPP)-conjugated synthetic peptide nucleic acids (PNAs) targeting the translational start site of the *Buchnera groEL* gene.^14^ This study extends the application of the CPP-conjugated PNA technique to target the *cls* gene in *Buchnera* to elucidate its function. We found that reducing *cls* expression resulted in decreased *Buchnera* titer and pronounced morphological abnormalities, underscoring the gene’s crucial role in cellular function. Furthermore, treated aphids exhibited delayed parturition, reduced fecundity, and mislocalization of *Buchnera* cells to their gut. These findings highlight the significance of the *cls* gene in maintaining *Buchnera*-host interactions, cellular integrity, and symbiont localization, advancing our understanding of this essential symbiosis.

## Results

### Design of peptide-conjugated anti-*cls* peptide nucleic acids

In our previous work on anti-*groEL* PNAs, we discussed the optimum choice of gene specific PNAs target region and sequence length.^14^ Similarly, the anti-*cls* PNAs were designed to be complementary to the region encompassing −5 to +5 bases from the translational start codon (ATG) of the *cls* gene (Figure 1C) (Table S1). To enhance the delivery of these PNAs into the bacteriocytes and, ultimately, into *Buchnera* cells, we conjugated them to the arginine-rich cell penetrating peptide (CPP), (RXR)_4_XB (Figure 1D). Previously, we found that PNA_mm serves as an ideal negative control, as it does not overlap with any translational start codon in either the *A. pisum* or *Buchnera* genomes. Hence, PNA_mm was used as the negative control in this study (Table S2).

### Anti-*cls* PNAs reduce *Buchnera cls* gene expression, leading to the reduction of *Buchnera* titer

**Figure 2:**
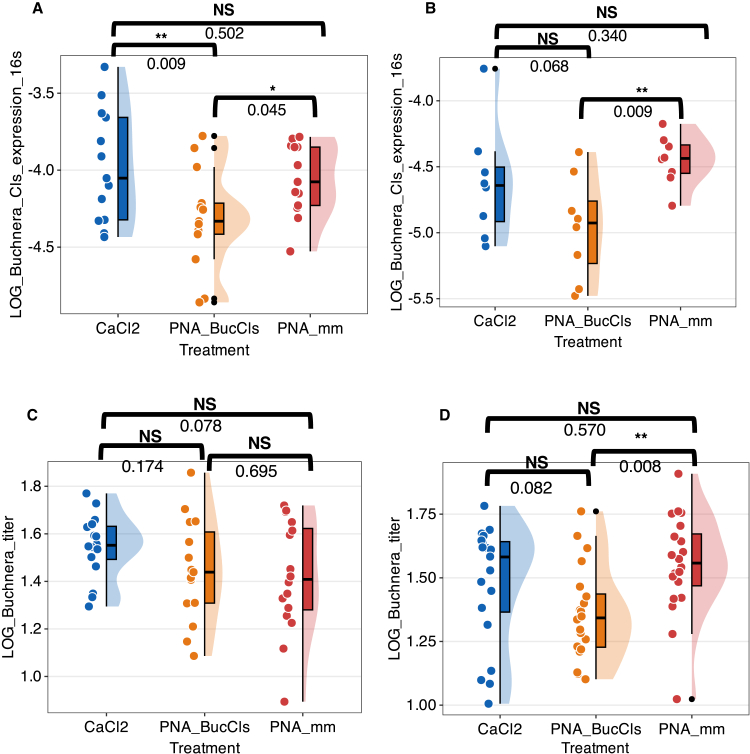
Effects of anti-*cls* PNAs on *Buchnera* gene expression and titer in aphid nymphs at 24 and 42 h post-treatment Panel A shows that aphid nymphs treated with PNA_BucCls (orange) exhibited significantly reduced the expression of the *cls* gene compared to the CaCl2 (blue) and PNA_mm (red) groups, with normalization to *Buchnera* 16S rRNA (*rrs* gene). Gene expression was quantified using RT-qPCR 24 h post-injection. Panel B shows that at 42 h, PNA_BucCls-treated nymphs again demonstrated significantly lower *cls* expression than the PNA_mm group, while a marginally significant difference was noted compared to the CaCl2 group. Panel C presents the comparative analysis of *Buchnera* titer in aphid nymphs 24 h post-injection. Panel D indicates that at 42 h post-injection, *Buchnera* titer in aphid nymphs treated with PNA_BucCls was significantly lower than that in the PNA_mm group, with a marginally significant difference observed between the CaCl2-treated group and the PNA_BucCls group. Data are shown as mean±*SD* for Panels A-C and median±*SD* for Panel D, with each dot representing one aphid nymph. Gene expression and titer data in Panels A-C were log-transformed and analyzed using one-way ANOVA followed by Fisher’s LSD test. Panel D was analyzed using the Kruskal-Wallis test with pairwise Wilcoxon rank sum post hoc comparisons. *p* < 0.05 (∗); *p* < 0.01 (∗∗); NS, not significant. See also Figures S1 and S2.

The functionality and significance of the sole remaining gene responsible for phospholipid metabolism in the *Buchnera* genome have long been elusive. To address this role, we injected peptide-conjugated PNAs specifically designed to target the *cls* gene. Gene expression of *cls* was quantified with RT-qPCR, using *Buchnera* 16S rRNA (*rrs*) for normalization (Figures 2A and 2B). The 5S rRNA gene was also used as a normalization control to ensure accuracy (Figures S1A and S1B). This supplementary normalization was necessary as the expression of *dnaK,* which is commonly used as a reference gene in *Buchnera* RT-qPCR assays*,* was affected by PNA_BucCls treatment (Figures S2A and S2B). Aphid nymphs injected with a 12 mM CaCl_2_ solution served as a negative control for comparison with the PNA_mm and PNA_BucCls treated groups.

First, the *cls* gene expression was evaluated using RT-qPCR 24 h after injection. A statistically significant reduction in *cls* mRNA levels was observed in PNA_BucCls-treated group compared to PNA_mm (Figure 2A). The CaCl2 treatment exhibited significantly higher *cls* expression compared to the PNA_BucCls treatment. However, no significant difference was found between the CaCl2-treated group and the PNA_mm-treated group. (CaCl2 = −3.98 ± 0.364 (mean±*SD*), PNA_BucCls = −4.31 ± 0.327, PNA_mm = −4.06 ± 0.233, *n* = 13; ANOVA, *F*(2, 36) = 4.13, *p* = 0.024)

To further evaluate long-term effects, we assessed *cls* expression at 42 h post-injection, with the concentration of PNAs increased to 15 μM (Figure 2B). The CaCl2-treated group showed a marginally significant difference from the PNA_BucCls-treated group. Again, the PNA_mm-treated group exhibited significantly higher *cls* gene expression compared to the PNA_BucCls-treated group. (CaCl2 = −4.62 ± 0.428 (mean±*SD*), PNA_BucCls = −4.96 ± 0.389, PNA_mm = −4.45 ± 0.191, *n* = 8; ANOVA, *F*(2, 21) = 4.35, *p* = 0.026). These results confirm the successful knockdown of *cls* in *Buchnera*.

We next investigated the impact of the interference of the *cls* gene on both *Buchnera* and the aphid host by examining changes in *Buchnera* titer. In a previous study, we demonstrated that treatment with anti-*groEL* PNAs reduced *Buchnera* titer in aphid nymphs within 24 h.^14^ However, in contrast to anti-*groEL* PNAs, PNA_BucCls treatment did not significantly lower *Buchnera* titer at 24 h compared to the CaCl2-and PNA_mm-treated groups (Figure 2C). The CaCl2-treated aphid nymphs did not exhibit significant differences between the PNA_mm and PNA_BucCls groups, nor was there a significant difference identified between aphid nymphs treated with PNA_mm and PNA_BucCls (CaCl2 = 1.55 ± 0.135 (mean±*SD*), *n* = 16, PNA_BucCls = 1.45 ± 0.216, *n* = 15, PNA_mm = 1.42 ± 0.235, *n* = 16; ANOVA, *F*(2, 44) = 1.79, *p* = 0.18).

However, extending the observation period to 42 h revealed a significant reduction in *Buchnera* titer in the PNA_BucCls-treated aphid nymphs (Figure 2D). Kruskal-Wallis test showed that *Buchnera* titer was significantly lower in PNA_BucCls-treated aphid nymphs compared to those treated with PNA_mm. The CaCl2-treated aphid nymphs showed a marginally significant difference in *Buchnera* titer compared to PNA_BucCls-treated aphid nymphs, while no significant difference was found between the CaCl2 and PNA_mm groups. (CaCl2 = 1.58 ± 0.232 (median±*SD*), PNA_BucCls = 1.34 ± 0.183, PNA_mm = 1.56 ± 0.195, *n* = 20; Kruskal-Wallis test, *H*(2) = 8.80, *p* = 0.012). Taken together, these results demonstrate that reducing *cls* gene expression via CPP-conjugated anti-*cls* PNAs, similar to anti-*groEL* PNAs, significantly impacts *Buchnera* titers over an extended period, underscoring the essential role of *cls* in *Buchnera*.^14^

### *cls* knockdown causes severe morphological abnormalities in *Buchnera* cells

**Figure 3:**
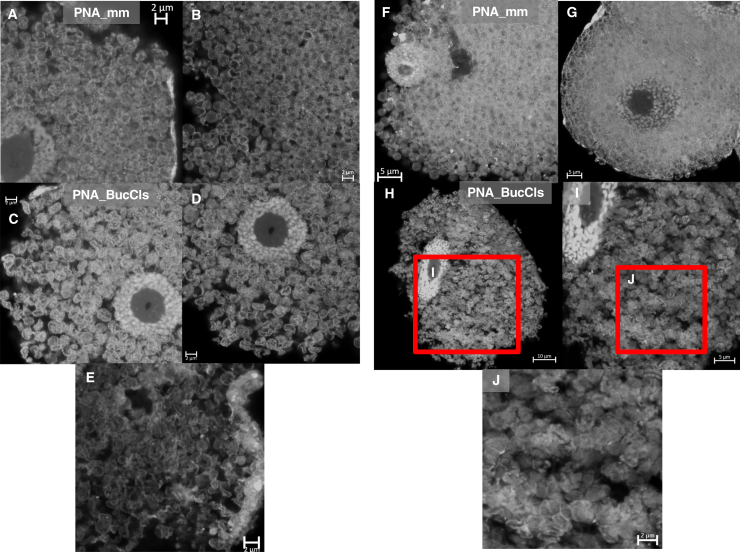
Super-resolution imaging of aphid bacteriocytes and *Buchnera* cells reveals PNA_BucCls effects at 24 and 40 h post-treatment Aphid nymphs were injected with either PNA_mm or PNA_BucCls at a concentration of 15 μM in a 12 mM CaCl_2_ solution. Following the respective incubation periods, the injected nymphs were dissected for analysis. Dissected bacteriocytes were fixed and stained with DAPI to visualize the nuclei of the bacteriocytes and the nucleoid of *Buchnera*. In the PNA_mm-treated group, normal *Buchnera* cells exhibited a characteristic round shape (Panels A-B). In contrast, distorted *Buchnera* cells were observed in the PNA_BucCls-treated aphid nymphs (Panels C-D). Zoom-in image shows that the *Buchnera* cells were curved and wavy under the PNA_BucCls treatment (Panel E). Similar methodologies were employed for the 40 h time point. *Buchnera* cells in the PNA_mm-treated group retained their typical round morphology (Panels F-G). However, as with the earlier time point, distorted *Buchnera* cells were again noted in the PNA_BucCls-treated aphid nymphs (Panels H-J). Zoom-in images show that the *Buchnera* cells form aggregates under the PNA_BucCls (Panels I-J). (Scale bars: A-E & J = 2 μm; F-G & I = 5 μm; H = 10 μm).

We next investigated the morphological phenotypes of *Buchnera* cells following anti-*cls* PNAs treatment using confocal or super-resolution microscopy. Bacteriocytes of aphid nymphs injected with 15 μM PNA_BucCls and PNA_mm were stained with DAPI and observed 24 h post injection (Figures 3A–3E). *Buchnera* cells were mostly spherical in shape in the bacteriocytes of PNA_mm-treated aphid nymphs (Figures 3A and 3B). On the other hand, *Buchnera* cells in the bacteriocytes of PNA_BucCls deviated discernibly from their typical round morphology, surprisingly, forming short tubular, wavy, and curved shapes (Figures 3C–3E), indicating severe morphological abnormalities.

To further characterize the morphological changes, the observation period was extended to 40 h post-injection (Figures 3F–3J). *Buchnera* cells in the bacteriocytes of PNA_mm-treated aphid nymphs were predominantly spherical (Figures 3F and 3G). In contrast, the PNA_BucCls-treated group exhibited severe morphological distortions (Figures 3H–3J). While some *Buchnera* cells displayed the short tubular or wavy cell morphology as in the 24 h post-treatment, most of the PNA_BucCls-treated *Buchnera* cells could hardly be identified as discrete individual cells 40 h post-treatment (Figure 3J). Instead, they formed clumps within the bacteriocytes (Figure 3J). The drastic morphological distortions following the *cls* knockdown underscore the crucial role of the *cls* gene in the normal structure and function of *Buchnera* cells.

### Knockdown of *Buchnera cls* severely impairs aphid fecundity and delays parturition

**Figure 4:**
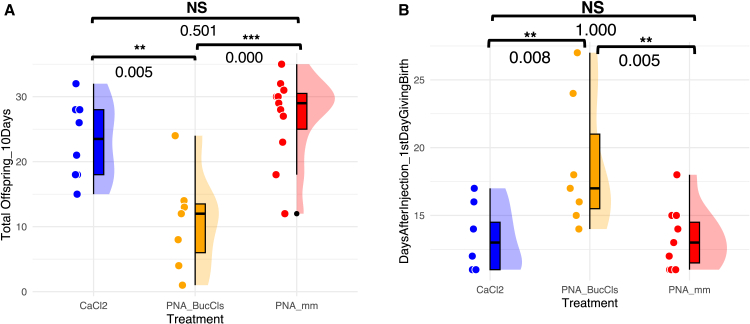
Fecundity and parturition timing of aphids treated with CaCl2, PNA_BucCls, and PNA_mm Second instar aphid nymphs were injected with 15 μM of peptide-conjugated anti-*cls* (PNA_BucCls; represented by the orange) or control PNAs (PNA_mm; represented by the red). An additional negative control group consisted of aphids treated with a 12 mM CaCl_2_ solution (blue). (A) The fecundity of aphids treated with PNA_BucCls was significantly lower than that of aphids in the CaCl2 and PNA_mm groups. (B) A significant delay in parturition was observed in the PNA_BucCls-treated aphid group compared to both the PNA_mm and CaCl2-treated groups. Data are shown as mean±*SD*, with each dot representing an individual treated aphid nymph. Statistical analyses were conducted using ANOVA (post hoc: Tukey’s HSD test). *p* < 0.01 (∗∗), *p* < 0.001 (∗∗∗); NS, not significant.

We investigated the impact on the host. Given that the *cls* knockdown caused a reduction in *Buchnera* titer, we speculate that aphid fecundity might be affected, as previous studies have demonstrated that aposymbiotic aphids suffer from a substantial reduction in reproduction.^15^^,^^16^ To test this, we measured the average number of offspring produced from the first day to the tenth day of parturition in aphids treated with PNAs or CaCl2. The fecundity of the three groups was analyzed using ANOVA (Figure 4A). No significant difference was found between the CaCl2-and PNA_mm-treated aphids. Notably, PNA_BucCls-treated aphids produced significantly fewer offspring than either the CaCl2-or PNA_mm-treated groups (CaCl2 = 23.2 ± 6.07 (mean±*SD*), *n* = 8, PNA_BucCls = 10.9 ± 7.54, *n* = 7, PNA_mm = 26.8 ± 6.71, *n* = 11; ANOVA, *F*(2, 23) = 12.38, *p* = 0.000).

In addition to reducing fecundity, the treatment of CPP-conjugated anti-*cls* PNAs also delayed the parturition in treated aphids (Figure 4B). The number of days post-injection until the first parturition was recorded and compared among CaCl2-, PNA_mm- and PNA_BucCls-treated aphids. The timing of parturition was similar between CaCl2-and PNA-mm treated aphids. However, PNA_BucCls-treated aphids experienced a marked delay in parturition (CaCl2 = 13.2 ± 2.38 (mean±*SD*), *n* = 8, PNA_BucCls = 18.7 ± 4.89, *n* = 7, PNA_mm = 13.3 ± 2.15, *n* = 11; ANOVA, *F*(2, 23) = 7.63, *p* = 0.003).

These observations indicate that knockdown of the *Buchnera cls* gene adversely affects the reproduction and development of the host insect. It remains unclear whether these effects are a direct consequence of *cls* gene interference or an indirect result stemming from the reduction in the *Buchnera* titer.

### Mislocalization of *Buchnera* to the aphid gut following anti-*cls* peptide nucleic acid treatment

**Figure 5:**
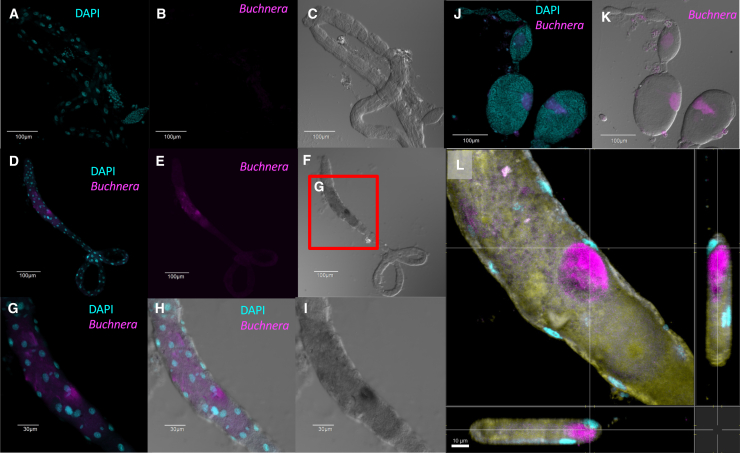
Unexpected detection of *Buchnera* cells in *A.pisum* gut. Aphid nymphs were injected with either PNA_mm or PNA_BucCls and subsequently dissected after 68 h The localization of *Buchnera* was assessed using a Cy5-conjugated ApisP2a. Panels A-C depicts the gut of an aphid treated with negative control, PNA_mm, which shows no detectable signals for *Buchnera*. In contrast, panels D-F illustrate the successful detection of *Buchnera* signals in the gut of *A. pisum*. Zoom-in images (G-I) reveal the aggregation of *Buchnera* cells within the gut. Positive control results (J-K) confirm the presence of *Buchnera* signals in the bacteriocytes of embryos from PNA_BucCls-treated aphid nymphs. Panel (L) presents the orthogonal views from z stack confocal imaging (z dimension = 0.29 μm per slice, 100 slices), further validating the detection of *Buchnera* signals in the aphid gut. Dissected guts were stained with DAPI to visualize cell nuclei (cyan), with *Buchnera*-specific FISH probe signals appearing in magenta and actin filaments visualized in yellow using phalloidin peptide. (Scale bars: A-F & J-K = 100 μm; G-I = 30 μm; L = 10 μm). See also Figures S3 and S4.

During the microscopic inspection of the aphids treated with anti-*cls* PNAs, we unexpectedly discovered *Buchnera* cells in an atypical location, the gut. Using a Cyanine 5 (Cy5)-conjugated *Buchnera*-specific probe, ApisP2a, we observed that Cy5 signals (magenta in Figure 5) were absent in the gut of PNA_mm-treated aphid nymphs (Figures 5A–5C), but the *Buchnera* signals were clearly visible in the gut of PNA_BucCls-treated aphid nymphs at 68 h post-injection (Figures 5D–5I). This is notable because *Buchnera* normally resides exclusively within bacteriocytes throughout the aphid life cycle, and to our knowledge, there have been no previous reports of *Buchnera* localization outside of the specialized cells.

To confirm the specificity of the probe, we included fluorescent-staining images of embryos as a positive control, where *Buchnera* signals were clearly identified within the bacteriocytes of embryos (Figures 5J and 5K). We further validated the presence of *Buchnera* in the gut by employing z stack imaging to generate orthogonal views (Figure 5L). *Buchnera* signals were distinctly observed within the gut, as evidenced by the detection of *Buchnera* signals situated between the nuclei of gut cells (stained with DAPI, cyan in Figure 5L). Additionally, the *Buchnera* signals were overlapped with phalloidin-stained actin filaments (yellow), confirming the localization of *Buchnera* within the gut tissue rather than merely surrounding it. We constructed another orthogonal view of z stack images that clearly illustrate the overlapping of *Buchnera* cells or bacteriocytes with the aphid gut (Figures S3A and S3B), which further clarifies that *Buchnera* cells indeed reside within the gut tissue itself, rather than merely adjacent to it.

We investigated whether this phenomenon also occurred at a different time point. Notably, we detected the signals of *Buchnera* in gut at a shorter post-treatment period, with *Buchnera* signals present in the gut of PNA_BucCls-treated aphid nymphs within 44 h post-injection (Figures S4A and S4B). *Buchnera* signals were absent in the gut of both PNA_mm and CaCl2-treated aphid nymphs (Figures S4C, S4D, S4E, and S4F). Likewise, the *Buchnera* signals were not detected in the aphid gut treated with PNA_GroEL dissected 48 or 68 h post-injection (Figures S4G, S4H, S4I, and S4J). Hence, the presence of *Buchnera* in the aphid gut was exclusively associated with PNA_BucCls treatment.

### Potential invasion of *Buchnera* cells into the aphid gut

**Figure 6:**
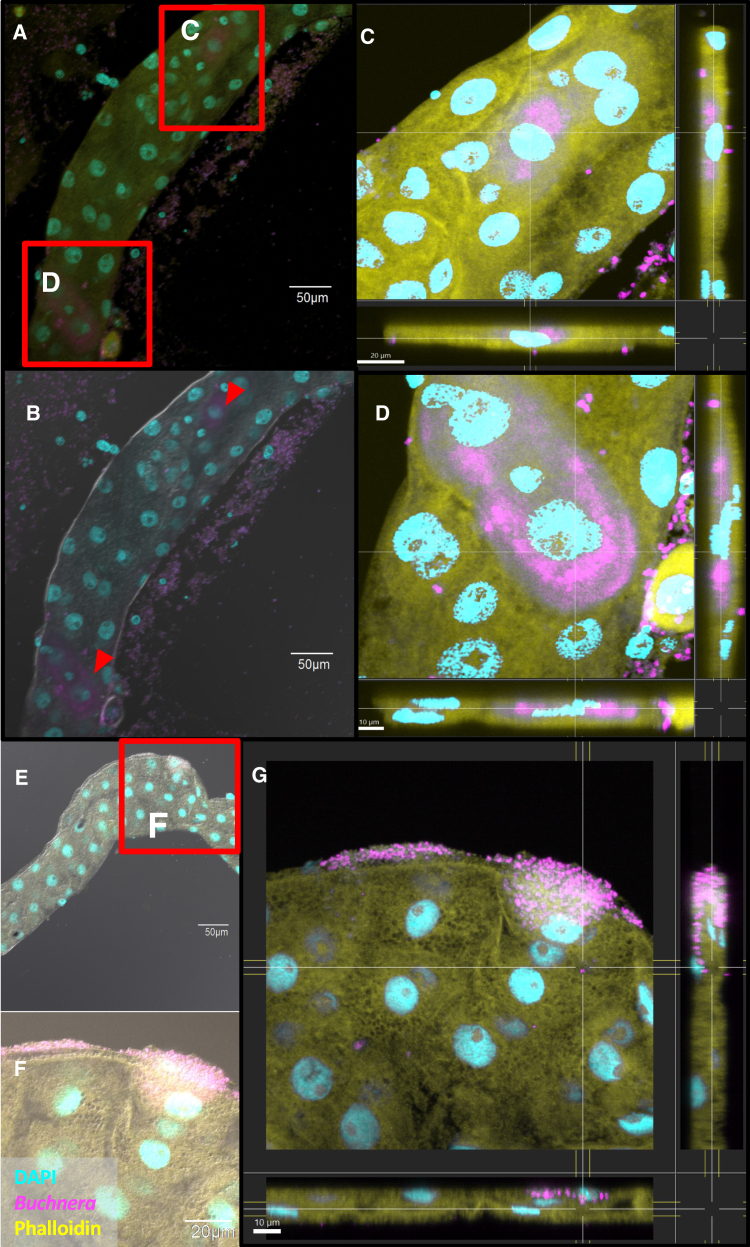
Localization and potential invasion of *Buchnera* cells in aphid gut Second instar aphid nymphs were injected with either PNA_BucCls (15 μM in a 12 mM CaCl_2_ solution) and subsequently dissected after 44 or 48 h. The localization of Buchnera was observed using a Cy5-labeled ApisP2a probe. Panels A and B illustrate the detection of *Buchnera* signals in two distinct regions of the aphid gut from a nymph treated with PNA_BucCls 48 h post-injection, indicated by red-colored arrowheads. Panels C and D provide orthogonal views derived from z stack confocal images (z-dimension = 0.22 μm per slice, 100 slices each for C and D), confirming the presence of *Buchnera* within the gut tissue. Panels E and F depict the presence of *Buchnera* cells predominantly at the periphery of the aphid gut, with indications of gradual invasion into the gut tissue as shown in panel G. This observation was further validated through orthogonal views obtained from z stack confocal imaging (z-dimension = 0.22 μm per slice, totaling 104 slices). Aphid nymphs were injected with either 15 μM PNA_BucCls, followed by a 44 h incubation period before dissection for images in E and F. Dissected gut tissues were stained with DAPI to visualize cell nuclei (cyan), with *Buchnera*-specific FISH probe signals represented in magenta and actin filaments visualized in yellow using phalloidin. (Scale bars: A-B & E = 50 μm; C & F = 20 μm; D & G = 10 μm). See also Figure S3.

An unexpected observation in anti-*cls* PNAs-treated aphid nymphs was the mislocalization of *Buchnera* into the gut, a compartment outside their typical intracellular habitat within bacteriocytes. Normally, *Buchnera* is strictly confined to specialized host cells and vertically transmitted from maternal bacteriocytes to developing embryos.^17^^,^^18^ In treated aphids, *Buchnera* cells were observed as concentrated clusters localized to the gut at multiple time points between 44 and 68 h post-injection. Among individuals in which *Buchnera* was detected in the gut, approximately 22.2% exhibited dual localization, with *Buchnera* signals appearing in two distinct regions of the same gut (Figures 6A–6D, S3C, and S3D). This observation suggests that *Buchnera* mislocalization may not always occur uniformly, although it remains unclear whether this reflects differences in timing, spatial microenvironmental factors (e.g., pH or immune activity), or both.

Moreover, we documented instances where *Buchnera* cells appear to be gradually invading aphid gut cells, with notable observations made at 44 h post-injection (Figures 6E–6G). While the presence of *Buchnera* within the gut cells may support the hypothesis of a potential invasion, we interpret these findings with caution. The biological significance of this mislocalization remains unclear and warrants further investigation.

### Impact of peptide nucleic acids on *Buchnera* genes involved in cell membrane synthesis, flagellar assembly, and cell fission

**Figure 7:**
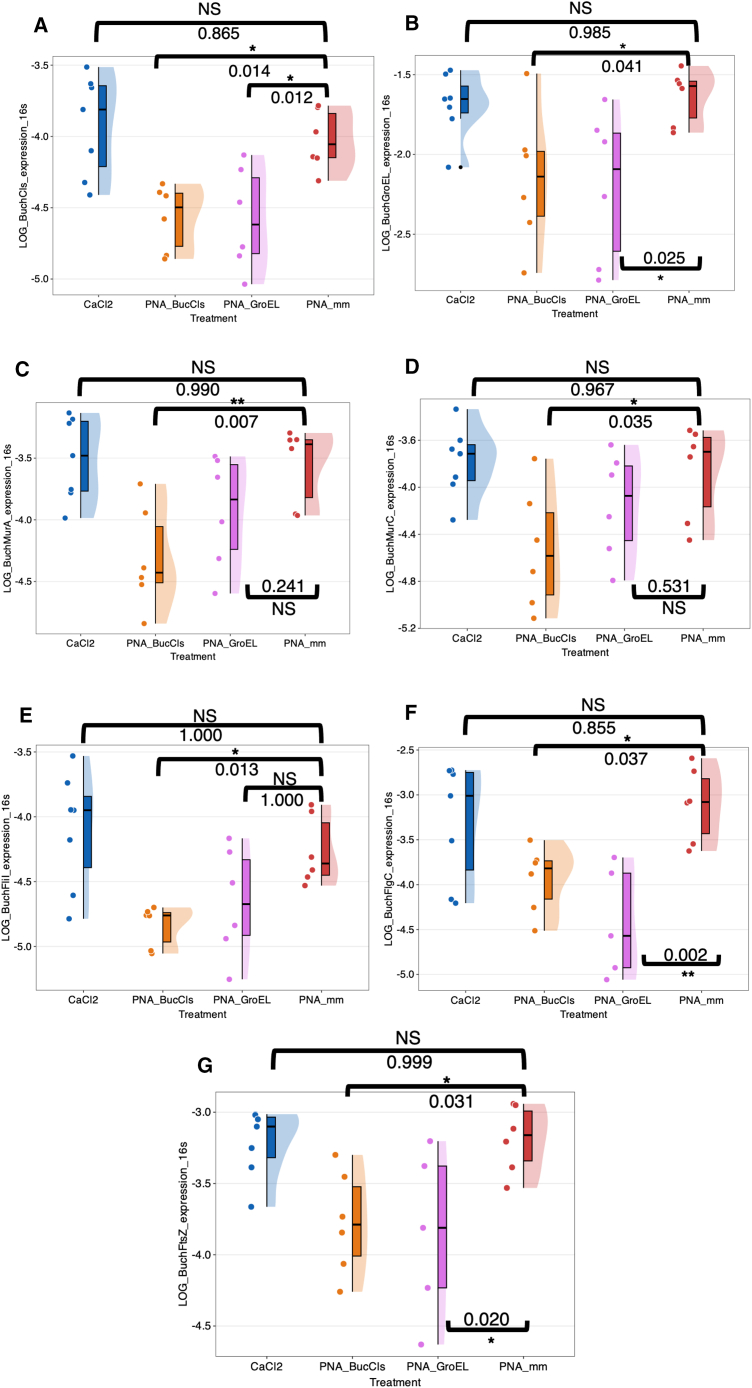
Expression analysis of genes involved in cell membrane synthesis, flagellar assembly, and cell fission Treatments include 10 μM PNA_BucCls (orange), PNA_mm (red), PNA_GroEL (pink), and 12mM CaCl_2_ (blue). All analyses used the same RNA samples. (A) *cls* expression was lower in PNA_BucCls and PNA_GroEL compared to PNA_mm, with no difference for CaCl2. (B) *groEL* expression was decreased in PNA_BucCls and PNA_GroEL compared to PNA_mm, with no difference for CaCl2. (C) *murA* expression was reduced in PNA_BucCls, but not in PNA_GroEL or CaCl2. (D) *murC* expression was lower in PNA_BucCls, but not in PNA_GroEL or CaCl2. (E) *fliI* expression was reduced in PNA_BucCls, but not in PNA_GroEL or CaCl2. (F) *flgC* expression was lower in both PNA_BucCls and PNA_GroEL, with no significant difference in CaCl2. (G) *ftsZ* expression was reduced in PNA_BucCls and PNA_GroEL compared to PNA_mm, with no significant difference in CaCl2. Data were analyzed using ANOVA (post hoc: Dunnett’s test), except for (E), which was analyzed by Kruskal-Wallis test (post hoc: pairwise Wilcoxon rank-sum test). Data are shown as mean±*SD* except for Panel E, where data are shown in median±*SD*. Each dot representing one aphid nymph. *p* < 0.05 (∗); *p* < 0.01 (∗∗); NS, not significant. See also Tables S3 and S4.

To further investigate how *cls* knockdown affects *Buchnera* beyond its immediate role in phospholipid metabolism, we analyzed the expression of several key genes associated with cell membrane synthesis (*murA* and *murC*), flagellar assembly (*fliI* and *flgC*), and cell fission (*ftsZ*) (Figures 7C–7G) (see also Tables S3 and S4 for statistical details). We employed PNA_GroEL as a control group for comparison. Besides, we also confirmed the effectiveness of both PNA_BucCls and PNA_GroEL treatments in knocking down their respective gene target prior to the gene expression analyses (Figures 7A and 7B). Notably, anti-*cls* PNAs treatment led to a reduction in the expression of all analyzed cell membrane synthesis- and flagellar assembly related genes, suggesting significant disruptions in *Buchnera* cell function. In contrast, anti-*groEL* PNAs treatment only reduced *flgC* expression compared to the control PNA_mm group, with no significant changes observed in *murA*, *murC*, or *fliI* expression (Figures 7C–7F). Additionally, we examined the expression of *Buchnera ftsZ*, which is crucial for z-ring formation during cell fission. Both PNA_BucCls and PNA_GroEL treatments resulted in reduced *ftsZ* expression (Figure 7G).

Overall, these results suggest that *cls* knockdown compromises essential genes involved in membrane integrity and cell division in *Buchnera*, providing insights into the mechanisms underlying the observed cellular aggregation and potential invasion into the aphid gut observed in the *cls* knockout aphids.

## Discussion

### Role of the *Buchnera cls* gene as revealed by gene interference experiments

The *Buchnera cls* is the sole remaining phospholipid synthesis-related gene in the *Buchnera* genome, representing a vital component for the study of endosymbiosis. The *Buchnera cls* gene is a homolog of the *Escherichia coli clsA* gene. The *E. coli clsA* gene encodes an enzyme that synthesizes cardiolipin (CL) by condensing two molecules of phosphatidylglycerol (PG).^10^^,^^19^ However, the function of the *cls* gene product in *Buchnera* remains uncertain, as some *Buchnera* strains, such as those in the aphid *Cinara cedri*, have completely lost this gene.^9^^,^^20^

This study is the first to investigate the function of the *cls* gene in *Buchnera* using the CPP-conjugated antisense PNAs method, which we previously developed for targeting the *Buchnera groEL* gene. Interference with the *cls* gene led to significant morphological abnormalities and a notable reduction in *Buchnera* titer, indicating its importance in *Buchnera*, at least in strains retaining this gene, such as *Buchnera* of *A. pisum*. While the exact molecular role of *cls* remains unexplained, our findings clearly demonstrate that its interference negatively impacts *Buchnera* cell viability.

Moreover, we also observed severe detrimental impacts on aphid reproduction and mislocalization of *Buchnera* in the gut, rather than bacteriocytes, in aphids treated with anti-*cls* PNAs. These results suggest that the *Buchnera cls* gene plays a crucial role in maintaining the aphid-*Buchnera* symbiosis.

### Retention of only *cls* in phospholipid synthesis is common among insect endosymbionts

Interestingly, the retention of only the *cls* gene in the phospholipid synthesis pathway is not unique to *Buchnera*. In fact, several other obligate insect endosymbionts exhibit a similar trend, wherein they lose most of the genes involved in phospholipid synthesis but retain a single *cls* gene. For example, in the whitefly (*Bemisia tabaci*) symbiosis, the endosymbiont *Portiera* has been shown to retain only the *cls* gene, as predicted through genome analysis and metabolic pathway reconstruction (KEGG data: mapno=00564+category=Portiera; https://www.genome.jp/pathway/mapno=00564&category=Portiera).^8^^,^^21^^,^^22^ Another remarkable example is found in the gammaproteobacterium *Moranella*, which resides in the cytoplasm of the betaproteobacterium *Tremblaya princeps* in the mealybug *Planococcus citri* (KEGG data: mapno=00564+category=Moranella; https://www.genome.jp/pathway/mapno=00564&category=Moranella).^8^^,^^23^ Similarly, *Mikella*, an enterobacterial symbiont of *Paracoccus marginatus*, shows the same pattern (KEGG data: mapno=00564+category=Mikella; https://www.genome.jp/pathway/mapno=00564&category=Mikella).^8^^,^^24^ These observations suggest that the retention of a minimal phospholipid synthesis capacity, centered on *cls*, may reflect the fundamental requirement to maintain a minimal level of cardiolipin synthesis for membrane integrity while the majority of the pathway becomes redundant in the host-provided environment.

### Delayed impact of anti-*cls* PNAs treatment on *Buchnera* titer

The reduction in *Buchnera* titer in the anti-*cls* PNAs-treated aphid nymphs was first observed at approximately 42 h post-treatment, which was a delayed response compared to the 24 h impact seen with anti-*groEL* PNAs treatment.^14^ This delay is noteworthy. According to existing literature, CL, an anionic phospholipid, is critical for cell fission and DNA replication.^12^^,^^13^ Despite reports of viable *E. coli*, a close relative of *Buchnera*, surviving without PG and CL, such viability is attributed to the presence of other anionic phospholipids such as phosphatidic acid (PA).^12^^,^^19^ This suggests that while CL depletion may initially be tolerable, its prolonged absence eventually leads to severe cellular dysfunction in *Buchnera*, which may explain the observed delay in titer reduction.

This delayed impact in *Buchnera* titer reduction following anti-*cls* PNAs treatment may also be caused by the specialized and stable intracellular environment provided by the symbiont-harboring bacteriocytes in aphids. The stable environment within bacteriocytes likely buffers *Buchnera* against the immediate impacts of CL depletion. However, over time, the inability to synthesize CL significantly impairs *Buchnera*, leading to a notable decrease in titer 42 h post-treatment. This observation aligns with the comparison to negative controls (CaCl2 and PNA_mm-treated aphid nymphs), where the PNA_BucCls group exhibited a significantly lower *Buchnera* titer at approximately 42 h post-treatment, reflecting the delayed yet eventual impact of CL depletion on *Buchnera* viability.

### Morphological alterations in *Buchnera* cells due to anti-*cls* PNAs treatment

Treatment with anti-*cls* PNAs resulted in notable morphological alterations in *Buchnera* cells. Unlike the wrinkled and crescent-shaped distortion observed with anti-*groEL* PNAs treatment,^14^ anti-*cls* PNAs induced a short tubular and curvy cell morphology (Figure 3E). Similar morphological changes were reported in the mitochondria of *Caenorhabditis elegans* upon the inhibition of the cardiolipin synthase (*crls-1*) gene.^25^ The eukaryotic *crls-1* gene product synthesizes CL using cytidine diphosphate-diacylglycerol (CDP-diacylglycerol) and phosphatidylglycerol (PG), whereas in bacteria such as *E. coli* and possibly *Buchnera*, ClsA produces CL through the condensation of two PG molecules. In *C. elegans*, knockdown of *crls-1* resulted in significantly elongated mitochondria, attributed to impaired membrane division due to the downregulation of the *crls-1* gene.

Although the morphological changes in *Buchnera* cells did not result in significant elongation, the transition from circular to short tubular and curvy forms may similarly reflect difficulties in membrane division caused by the *cls* gene downregulation. In addition, *Buchnera* cells became indistinguishable as individual cells around 42 h post-treatment, further suggesting a substantial impact of *cls* interference on cell division.

### Impact of anti-*cls* peptide nucleic acid treatment on aphid fecundity and parturition timing

The effects of *cls* knockdown extend beyond mere morphological abnormalities in *Buchnera* and impact the reproductive success of the host insect. The fecundity of aphids treated with PNA_BucCls was notably reduced compared to the CaCl2-and PNA_mm-treated groups. Additionally, the timing of parturition in PNA_BucCls-treated aphids was significantly delayed relative to the control groups. These findings suggest that while PNA_BucCls treatment does not immediately impact host survival, as indicated by the survival analysis where no significant differences were observed among the three treatment groups, it does negatively affect reproductive aspects such as fecundity and parturition timing (Figure S5).

The observed reduction in fecundity can be attributed to a decrease in *Buchnera* titer. Previous studies have demonstrated that aposymbiotic aphids, which lack their symbiotic *Buchnera* bacteria, suffer substantial reductions in reproductive rates.^15^^,^^16^ In this context, the delay in parturition and reduced fecundity in PNA_BucCls-treated aphids are likely to result from diminished nutrient contributions, particularly essential amino acids, provided by *Buchnera* to the host.

Environmental factors such as plant drought stress and nitrogen fertilization can also influence aphid reproduction.^26^^,^^27^ However, since the aphids in this study were raised under controlled and consistent conditions, the delayed parturition observed in anti-*cls* PNAs-treated aphids is best attributed to the reduction in *Buchnera* titer. While we cannot entirely exclude the possibility that anti-*cls* directly impacted the aphid reproduction, our findings suggest that its impact is primarily mediated through symbiont depletion.

### Unexpected localization of *Buchnera* in *A. pisum* gut

An unexpected yet critical observation in anti-*cls* PNAs-treated aphid nymphs was the localization of *Buchnera* in the gut. While previous studies have reported the detection of symbiont proteins, particularly molecular chaperones such as GroEL, in the aphid gut, the detection of *Buchnera* cells in this region is highly unusual.^28^^,^^29^ GroEL has been shown to act as a receptor for the garlic lectin, facilitating its toxic effects on aphids. However, the observation of *Buchnera* cells within the gut, a compartment outside their typical intracellular niche in bacteriocytes, suggests a potential deviation from the normally restricted localization patterns seen in these symbionts.

In anti-*cls* PNAs-treated aphid nymphs, *Buchnera* cells were distinctively observed as concentrated clusters within the gut. Interestingly, these clusters appeared in different regions within the gut at multiple time points, including 44, 48, and 68 h post-injection. Despite our best efforts, *Buchnera* cells were not detected in the control groups treated with mismatched PNAs (PNA_mm) or the CaCl2 solution, as well as in aphid nymphs treated with anti-*groEL* PNAs, reinforcing the idea that this mislocalization of *Buchnera* is specifically induced by anti-*cls* PNAs treatment. Additionally, the close proximity of the gut and bacteriocytes may also explain why *Buchnera* mislocalized to the gut but not to other tissues.

These findings prompt closer examination of how *Buchnera* localization is maintained within host tissues and whether such perturbations could influence vertical transmission. While our observations point to disrupted intracellular residency following *cls* knockdown, it remains to be determined whether these effects persist across developmental stages or generations.

Under normal conditions, *Buchnera* is strictly confined to bacteriocytes, and vertical transmission to stage-seven embryos is thought to be the only natural route through which the symbiont exits this intracellular niche.^17^^,^^18^ The disruption of *Buchnera* membrane integrity, as seen following *cls* knockdown, appears to disturb this normally restricted localization. It is plausible that host surveillance mechanisms, such as the immune recognition of altered bacterial surface properties, contribute by detecting compromised *Buchnera* cells and promoting their expulsion into the gut. Host-mediated recognition and control of symbionts have been described in other symbiotic systems.^30^^,^^31^^,^^32^ However, the extent to which host responses versus bacterial defects contribute to *Buchnera* mislocalization remains unclear and requires further investigation.

### The impact of anti-*cls* peptide nucleic acid treatment on *Buchnera* cell membrane synthesis, flagellar assembly, and cell division

Given the molecular function of Cls homologs in bacterial membrane synthesis, its disruption likely compromises the structural integrity of the *Buchnera* cell membrane. This hypothesis is supported by the significant downregulation of several genes involved in bacterial membrane biosynthesis and flagellar assembly, such as *murA*, *murC*, *fliI*, and *flgC*, observed in the PNA_BucCls-treated group compared to the negative control (PNA_mm-treated group) (Figures 7C–7F). This downregulation suggests a broader impact of *cls* interference on *Buchnera* membrane assembly and integrity.

To investigate whether the *cls* gene interference also affects cell division, we examined the expression of *Buchnera ftsZ*, a gene essential for Z-ring formation during bacterial fission. Reduced *ftsZ* expression was observed in both PNA_BucCls- and PNA_GroEL-treated groups, suggesting a potential disruption of cell division processes. Impaired cell fission, in conjunction with membrane disintegration, may contribute to the mislocalization of *Buchnera* cells in the gut.

Building on these findings, we speculate that disruptions to *Buchnera* membrane integrity could alter its recognition or stability within host tissues. Such changes might influence how the host interacts with compromised symbionts, potentially affecting intracellular maintenance or clearance. At present, we cannot determine whether the *Buchnera* cells observed in the gut are viable, damaged, or undergoing degradation. Future studies should assess the physiological state of these mislocalized cells and explore whether specific bacterial or host factors influence their localization.

### Effects of anti-*cls* peptide nucleic acids on the expression of *Buchnera* stress response and off-target interactions with host genes

The observed reduction in *Buchnera dnaK* expression raises intriguing questions about the interplay between membrane integrity, stress responses, and overall cellular homeostasis in *Buchnera*. As a molecular chaperone, DnaK plays a critical role in protein folding and responding to cellular stress.^33^^,^^34^ Under typical stress conditions, *dnaK* expression is generally upregulated; however, in our study, *cls* knockdown reduced *dnaK* expression. This reduction may indicate impaired protein folding and stress response capabilities. One possible explanation is that the disruption of *cls*, a gene essential for cardiolipin (CL) biosynthesis, severely compromised membrane integrity. CL is a key component of bacterial membranes, vital for maintaining membrane structure and supporting processes such as protein localization, nutrient transport, and energy metabolism.^35^^,^^36^ Severe membrane damage may have impaired *Buchnera*’s ability to mount a typical stress response, leading to the downregulation of *dnaK* as a consequence of catastrophic cellular dysfunction.

Due to constraints in PNAs length during design, the anti-*cls* PNAs exhibited sequence overlaps at the translational start sites of four *A. pisum* genes: LOC107882452 (Apisum_RT), LOC100158964 (*kptn*/kaptin), LOC115034171, and LOC103310598 (Table S5). The potential off-target effects on these two host genes were analyzed in this study (Figures S2D and S2E). Despite these target sequence matches, the expression levels of *Apisum_RT* and *kptn/kaptin* did not significantly differ from the control group treated with PNA_mm. In contrast, analysis of the same RNA samples revealed a significant reduction in *Buchnera cls* expression in the PNA_BucCls-treated group compared to the PNA_mm-treated group (Figure S2C). These findings suggest that the anti-*cls* PNAs had minimal off-target effects on the aphid genes tested, thereby validating their specificity toward *Buchnera* genes and highlighting the precision of PNAs as tools for gene interference in unculturable bacterial symbionts.

This study examined the function of the *cls* gene in *Buchnera*, the sole remaining gene responsible for phospholipid metabolism in its highly streamlined genome. Targeted interference with the *cls* gene using the PNAs technique resulted in compromised *Buchnera* integrity, as evidenced by decreased titer and severe morphological abnormalities. These findings demonstrate the essential role of the *cls* gene in the *Buchnera* cell. The disruption of *Buchnera* correlated with impaired aphid reproduction, manifesting as reduced fecundity and delayed parturition. These host phenotypic alterations underscore the pivotal role of the *Buchnera cls* gene for the integrity of both the bacterial symbionts and the insect host. Collectively, our results highlight the critical importance of the *cls* gene in maintaining the symbiosis between aphids and *Buchnera*.

A notable observation in this study is the mislocalization of *Buchnera* cells in the aphid gut following *cls* knockdown, deviating from their typical confinement within bacteriocytes. This mislocalization may be associated with compromised *Buchnera* membrane integrity, potentially resulting from disrupted cardiolipin biosynthesis. Such membrane destabilization could interfere with the ability of the symbiont to maintain intracellular residency. These findings suggest new directions for exploring the molecular and cellular processes that govern *Buchnera* localization within host tissues. While the underlying cause remains to be fully understood, both bacterial membrane defects and possible host responses to altered symbiont physiology may contribute. Further investigation is needed to identify the bacterial determinants and host regulatory mechanisms that ensure proper *Buchnera* positioning and transmission fidelity.

### Limitations of the study

This work provides key insights into the role of the *cls* gene in *Buchnera* using a PNAs knockdown method, but a few caveats should be noted. First, the delivery and uptake of PNAs may vary between individuals and tissues, potentially leading to uneven knockdown efficiency. Second, although we carefully designed PNAs for high target specificity, we cannot fully rule out low-level off-target effects on non-*cls* transcripts or host RNAs due to the short length constraints of PNAs. Third, while we observed *Buchnera* mislocalization to the gut, additional assays are needed to confirm whether these cells remain viable and transmissible. Lastly, although the reduction in *cls* gene expression coincides with *Buchnera* mislocalization, further studies are needed to clarify the causal relationship and to elucidate the underlying mechanisms, including the specific molecular functions of *cls*.

## Resource availability

### Lead contact

Further information and requests for resources and reagents should be directed to and will be fulfilled by the lead contact, Shuji Shigenobu (shige@nibb.ac.jp).

### Materials availability

This study did not generate new, unique reagents.

### Data and code availability

• All custom R scripts used for statistical analyses have been deposited at Zenodo: https://doi.org/10.5281/zenodo.14695382.
• Any additional information reported in this study is available from the lead contact upon reasonable request.

## Acknowledgments

This work was funded by the Japan Society for the Promotion of Science (JSPS) KAKENHI Grants-in-Aid for Scientific Research (A) (No. JP20H00478 and JP24H00580) to S. S. We would also like to express our earnest gratitute toward all members of the Laboratory of Evolutionary Genomics for their advice. We thank the Optics and Imaging Facility, NIBB Trans-Scale Biology Center, for their technical support.

## Author contributions

K. T. and S. S. designed the study; K. T. performed the experiments and analyzed the data; K. T. wrote the article, and K. T. and S. S. contributed to its revision.

## Declaration of interests

The authors declare no competing interests.

## STAR★Methods

### Key resources table

**Table:** 

REAGENT or RESOURCE	SOURCE	IDENTIFIER
**Bacterial and virus strains**		

*Buchnera aphidicola* strain ApL	This study	N/A

**Chemicals, peptides, and recombinant proteins**		

Alexa Fluor™ 488 phalloidin	Thermo Fisher Scientific	Cat#A12379
DAPI	Dojindo Laboratories	Cat#340-07971
Proteinase K	Qiagen	Cat#19131

**Critical commercial assays**		

Takara Nucleospin RNA Plus XS RNA extraction kit	Takara Bio Inc.	Cat# 740990.50
Takara One Step TB Green PrimeScript™ RT-PCR kit	Takara Bio Inc.	Cat# RR066A
KOD SYBR® qPCR Mix	Toyobo Co.	Cat# QKD-201

**Experimental models: Organisms/strains**		

*Acyrthosiphon pisum* strain ApL parthenogenetic clonal N2 nymphs, maintained at the National Institute for Basic Biology, Aichi, Japan	Kanbe and Akimoto^37^	N/A

**Oligonucleotides**		

For primer sequences, please see Table S6		
Cyanine 5-conjugated ApisP2a (Buchnera FISH probe)	Koga, Tsuchida and Fukatsu^38^	
Anti-*cls* PNAs (PNA_BucCls)	This study	
Anti-*groEL* PNAs (PNA_GroEL)	Tan and Shigenobu^14^	
Control PNAs (PNA_mm)	Tan and Shigenobu^14^	

**Deposited data**		

Custom R scripts for statistical analyses	This study	https://doi.org/10.5281/zenodo.14695381
Multiple sequence alignment & phylogeny code	This study	https://doi.org/10.5281/zenodo.14695381

**Software and algorithms**		

Muscle (Ver 5.1)	Edgar^39^	https://drive5.com/muscle5/
RAxML-NG (Ver 0.9.0)	Kozlov et al.^40^	https://github.com/amkozlov/raxml-ng
Primer3Plus (Ver 2.6.1)	Untergasser et al.^41^	https://www.bioinformatics.nl/cgi-bin/primer3plus/primer3plus.cgi
ZEN software (Blue edition; Ver. 3.4.91)	ZEISS AG	https://www.micro-shop.zeiss.com/en/es/softwarefinder/software-categories/zen-blue/
FV10-ASW (Ver. 3.0)	Olympus Europa Holding GmbH	https://evidentscientific.com/en/downloads?product=FV10-ASW
IMARIS X64 (Ver. 9.7.1)	Oxford Instruments Group	https://imaris.oxinst.com/
R (Ver 4.2.2)	R Core Team^42^	https://www.r-project.org/
Devtools (Ver. 2.4.5)	Wickham et al.^43^	https://devtools.r-lib.org/
DescTools (Ver. 0.99.50)	Signorell^44^	https://andrisignorell.github.io/DescTools/
dplyr (Ver. 1.1.4)	Wickham^45^	https://github.com/tidyverse/dplyr
ggpubr (Ver. 0.6.0)	Kassambara^46^	https://github.com/kassambara/ggpubr
smplot2 (Ver. 0.1.0)	Min and Zhou^47^	https://github.com/smin95/smplot2
survival (Ver. 3.5–7)	Therneau^48^	https://github.com/therneau/survival
survminer (Ver. 0.5.0)	Kassambara, Kosinski and Biecek^49^	https://github.com/kassambara/survminer
ggplot2 (Ver. 3.4.4)	Wickham and Sievert^50^	https://github.com/tidyverse/ggplot2
ggsurvfit (Ver. 1.0.0)	Sjoberg et al.^51^	https://github.com/pharmaverse/ggsurvfit

**Other**		

Nippi-sterilized Biomasher II	Funakoshi Co.	Cat# 893062
Roche LightCycler® 96 real-time PCR instrument	Roche Applied Science	Cat# 05815916001

### Experimental model and study participant details

#### Aphids

This study used the parthenogenetic clone of the pea aphid *A. pisum* str. ApL (also known as Sap05Ms2) obtained from Sapporo, Hokkaido, Japan,^37^ which is maintained at the National Institute for Basic Biology, Aichi, Japan. Viviparous aphids were maintained on broad bean plants (*Vicia faba* L.) at 16°C with a photoperiod of 16/8 h light/dark.

### Method details

#### Phylogeny analysis of *Buchnera cls* gene

Multiple sequence alignment was performed using Muscle (Ver 5.1) with default setting.^39^ Maximum likelihood phylogenetic tree inference was conducted using RAxML-NG software (Ver 0.9.0) starting with 10 parsimony-based trees (--tree pars{10}), model LG + G8+F (--model LG+G8+F) and 200 bootstraps (--bs-trees 200) (Ver. 0.9.0).^40^ Amino acids sequences of cardiolipin synthase genes including GenBank: XP_003243782.1, WP_011053787.1, NP_415765.1, NP_415310.1, NP_415564.2, AHG61261.1, NP_001262969.1, NP_651418.1, NP_733116.1, NP_733117.1, BAB12983.1, NP_010139.1 were obtained from National Center for Biotechnology Information (NCBI).^52^

#### Aphid rearing

This study used the parthenogenetic clone of the pea aphid *A. pisum* str. ApL (also known as Sap05Ms2) obtained from Sapporo, Hokkaido, Japan.^37^ Viviparous aphids were maintained on broad bean plants (*Vicia faba* L.) at 16°C with a photoperiod of 16/8 h light/dark. Four viviparous adult female aphids were randomly selected and placed in culture cups containing young broad bean plants. The adult female aphids were allowed to reproduce for 48 h before being removed from the culture cup. The offspring aphid nymphs in each culture cup were then allowed to undergo a 48 h growth phase. Second instar stage (N2) aphid nymphs were used in the experiment.

#### Design of peptide-conjugated antisense PNAs

The important features for preparing PNAs were written in the results section. To obtain the *Buchnera cls* gene sequences, *Buchnera aphidicola* str. APS (GenBank accession number: NC_002528.1; 298238 bp – 299698 bp for *cls* gene) was used.^9^ Peptide-conjugated anti-*cls* PNAs (PNA_BucCls) and control PNAs, referred to as PNA_mm, were designed (Table S2).

To ensure specificity, BLAST was used to identify potential non-specific target sites for the PNAs sequences. For detecting nonspecific binding sites and assisting in the design of PNAs targeting a gene’s translational start site, a custom Python script was developed, available through the Google Colab notebook.^14^ The reference *A. pisum* genome sequence GCF_005508785.2 was utilized alongside the *Buchnera aphidicola* strain APS genome (GenBank accession number: NC_002528.1) to identify non-specific binding sites of PNAs.

#### Microinjection

We employed the microinjection technique following the protocol outlined in Tan and Shigenobu^14^ Details on capillaries preparation and injection setting were stated in the cited publication.

#### The effect of antisense peptide conjugated PNAs on *Buchnera* genes expression

Individual aphid nymphs from the same clonal line were independently injected with either 10 μM PNA_BucCls or PNA_mm. Group CaCl2 included aphid nymphs injected with 12 mM CaCl_2_ solution was added as negative control. Following the injection, the treated aphid nymphs were housed in plastic cases, each containing a broad bean seedling. To examine the effect of peptide-conjugated anti-*cls* PNAs on the expression of *Buchnera cls* genes, RNA was extracted from the injected aphid nymphs 24 h post-injection using the Takara Nucleospin RNA Plus XS RNA extraction kit (Takara Bio Inc., Shiga, JP). For RNA extraction, a single injected aphid nymph was homogenized with a Nippi-sterilized Biomasher II (Funakoshi Co., Ltd., Tokyo, JP) using Lysis Buffer I from the kit. RNA was extracted according to the manufacturer’s instructions. The same procedure was used to extract RNA from aphid nymphs treated for 42 h post-injection with 15 μM PNA_BucCls, PNA_mm, and 12 mM CaCl_2_.

#### The effect of anti-*cls* PNAs to *Buchnera* titer in the pea aphid

Individual aphid nymphs were injected with 15 μM of PNA_BucCls or PNA_mm. Additional negative control group, CaCl2 included aphid nymphs injected with 12 mM CaCl_2_ solution. The injected aphid nymphs were kept in a plastic case containing broad bean seedlings. At 24 h post-injection, DNA was extracted from the injected aphid nymphs. A single injected aphid nymph was homogenized with a Nippi-sterilized Biomasher II (Funakoshi Co., Ltd., Tokyo, JP) in a tube containing 30 μl Buffer A (10 mM Tris-HCl, 1 mM EDTA, 25 mM NaCl in UltraPure distilled water) with 0.4 mg/ml Proteinase K (QIAGEN N.V., Venlo, NL). Homogenized samples were incubated at 37°C for 1.5 h to lyse cell, followed by 98°C for 2 min to denature proteinase K. The extracted DNA samples were carefully stored in a freezer at −20°C for subsequent analysis. The DNA samples were used to assess the effect of peptide-conjugated anti-*cls* PNAs on *Buchnera* titer in pea aphids. The same procedure was used to assess the impact of anti-*cls* PNAs on *Buchnera* titer at 42 h post-injection.

#### Reverse transcription-quantitative polymerase chain reaction (RT-qPCR)

The expression levels of *Buchnera* and *A. pisum* genes were evaluated using RT-qPCR assays by the Takara One Step TB Green PrimeScript RT-PCR kit (Takara Bio Inc., Shiga, JP) and the Roche LightCycler 96 real-time PCR instrument (Roche Applied Science, Penzberg, DE). Each RNA sample was extracted from a single injected aphid, with each sample representing one biological replicate. The sample sizes are provided in the results section, and the experiments were conducted across four independent experimental batches. The RT-qPCR procedure was initiated with a single round of reverse transcription at 42°C for 5 min, followed by denaturation at 95°C for 10 s. Subsequently, 35 cycles of PCR reactions were performed, involving denaturation at 95°C for 5 s, followed by annealing and extension at 60°C for 20 s. Lastly, a 3-step melting curve analysis was applied, starting with 95°C for 10 s, followed by 65°C for 60 s and finally 97°C for 1 s. Gene amplification for *Buchnera* and *A. pisum* genes were carried out using the primers listed in Table S6. Primers were design using Primer3Plus (Ver 2.6.1).^41^ For normalization in gene expression analyses, *Buchnera* gene expression was normalized against the *Buchnera* 16S rRNA (*rrs*) or 5S rRNA genes, while the *A. pisum rpL7* gene was used for *A. pisum* gene expression normalization (Table S6).^53^ Log-transformation of gene expression values was performed prior to statistical analyses.

#### Quantitative polymerase chain reaction (qPCR)

To quantify *Buchnera* titer in aphid nymphs treated with CaCl2, PNA_BucCls and PNA_mm, the qPCR with KOD SYBR® qPCR Mix (Toyobo Co., Ltd., Osaka, JP) using Roche LightCycler® 96 real-time PCR instrument (Roche Applied Science, Penzberg, DE) was performed. Each DNA sample was extracted from a single injected aphid, with each sample representing one biological replicate. The sample sizes are provided in the results section, and the experiments were conducted across four independent experimental batches. The qPCR assay began with pre-incubation at 98°C for 2 min followed by a 2-step amplification process. During this amplification, the DNA was denatured at 98°C for 20 s, and then subjected to touchdown annealing from 66°C to 57°C, with a temperature decrement of 3°C at each step, for 10 s. Subsequently, a 3-step amplification was applied, involving denaturation at 98°C for 20 s, annealing at 66°C for 10 s, and extension at 68°C for 30 s. The 3-step amplification was conducted for 40 cycles. Lastly, a 3-step melting curve analysis was applied, starting with 95°C for 10 s, followed by 65°C for 60 s and finally 97°C for 1 s. For the qPCR assay, the BuchDnaK_F1018 and BuchDnaK_R1142 primers, which specifically targeted the *dnaK* gene of *Buchnera* were utilized. The rpL7_F and rpL7_R primers were used to amplify the *A. pisum rpL7* gene, which served as a normalization reference for the relative quantification of *Buchnera dnaK* gene.^53^ The relative abundance of *Buchnera dnaK* gene served as an estimation for *Buchnera* titer for each treated aphid nymphs. Log-transformation was applied to the *Buchnera* titer for subsequent statistical analyses.

#### Sample preparation for super-resolution laser scanning confocal microscopy imaging

The sample preparation for super-resolution laser scanning was applied as described previously in Tan and Shigenobu^14^ to observe any morphological changes in *Buchnera* cells.^14^

A Zeiss LSM 980 with Airyscan 2 in Airyscan SR mode was used to obtain single optical plane images. Visualization was performed by fluorescence and differential interference contrast (DIC) microscopy (Zeiss AG, Oberkochen, DE). Images were taken with a Plan-Apochromate 63x/1.40 oil immersion lens. Images were processed using ZEN software in the default 2D super-resolution mode (Blue edition; Ver. 3.4.91). The imaging channels for Airyscan were set as follows: λex = 473 nm (0.2%, detector gain = 750 V) and detection wavelength = 380–735 nm for DAPI; λex = 488 nm (0.2%, detector gain = 350 V) and detection wavelength = 300–900 nm for DIC. Linear unmixing was performed using the Automatic Component Extraction technique, a built-in function of the ZEN software, to remove autofluorescence.

#### Fluorescent *in situ* hybridization

Guts were dissected from aphid nymphs injected with 15 μM PNA_BucCls, PNA_mm, PNA_GroEL in 12 mM CaCl_2_ solution. Aphid nymphs treated solely with 12 mM of CaCl_2_ solution was added as negative control. Dissections were conducted at 44, 48 and 68 h post-injection. Aphid guts were dissected out from aphid body in 60% ethanol (EtOH) and allowed to get partially dried on glass slide. The gut samples were fixed using a 4% paraformaldehyde phosphate buffer solution (Fujifilm Wako Pure Chemical Corp., Osaka, JP) for 30 min. Following fixation, the gut tissues were washed twice with ice-cold 1x Phosphate buffered saline (PBS) (Gibco 10 x PBS pH 7.4, Thermo Fisher Scientific, MA, USA) added with 0.2% Triton® X-100 (Sigma-Aldrich®, Burlington, MA, USA) (Ptx) for 10 min each. To prepare for staining, the tissues were washed twice with hybridization buffer (HYB) (20 mM Tris-HCl, 0.9 M NaCl, 0.01% SDS, and 30% (v/v) formamide) for 5 min each. After the HYB wash, the tissues were homogenized with HYB at 37°C for an hour. A HYB staining solution containing 1 μg/ml 4,6-diamidino-2-phenylindole (DAPI) to stain DNA, 100 nM Cyanine 5 (Cy5)-conjugated ApisP2a (5′-CCTCTTTTGGGTAGATCC-3′) for *Buchnera* observation and 2 U/ml of Alexa Fluor ™ 488 phalloidin probe for filamentous actin staining was prepared.^38^ The tissues were stained overnight at 37°C.

After the overnight staining, the tissues were washed thrice using Ptx for 10 min each. Then, the stained tissues were mounted on glass slides with VECTASHIELD® Antifade Mounting Media (Funakoshi Co., Ltd., Tokyo, JP). The slides were observed using an Olympus FluoView FV1000 confocal microscope (Olympus, Tokyo, Japan) under UPLSAPO 20X NA:0.75 or UPLSAPO 60X W NA:1.20 objective lens. For DAPI imaging, the imaging channel was set at λex = 405 nm and λem = 430–455 nm, while DIC microscopy was conducted using a 473 nm laser. The phalloidin (Alexa Fluor™ 488) imaging channel was set at λex = 473 nm and λem = 490–590 nm while *Buchnera* probe (Cy5) imaging channel was set at λex = 635 nm and λem = 655–755 nm.

Nine aphid nymphs showing gut-localized *Buchnera* were imaged across six independent experiments conducted at 44, 48 and 68 h post-injection. For z-stack imaging, images were taken at a resolution of 512 × 512 pixels. Step size and the number of optical slices were mentioned in the figure captions. After acquisition, images were processed using FV10-ASW (Ver. 3.0) software. Orthogonal views of aphid gut images were prepared by IMARIS X64 (Ver. 9.7.1) software.

#### Analysis of aphid fecundity and parturition timing

Fecundity and parturition timing of second instar aphid nymphs injected with 15 μM peptide-conjugated antisense *cls* PNAs (PNA_BucCls, *N* = 7) or control PNAs (PNA_mm, *N* = 11) were assessed. Additional negative control group, CaCl2 included aphid treated with 12 mM CaCl_2_ solution (*N* = 8). The treated aphid nymphs were housed in plastic cases, with eight to ten aphids per container, each containing a broad bean seedling. Aphids that survived 10 d post-injection were individually transferred into separate plastic containers to observe the timing of parturition and quantify the number of offspring. The total number of offspring was determined by counting the number of nymphs born from the first day of parturition until the 10th day. The timing of parturition was recorded as the number of days after injection that the aphid first reproduced. The maximum observation period for parturition timing extended to 27 d post-injection.

#### The effect of peptide conjugated PNAs on untargeted *Buchnera* and *A. pisum* gene expression

RT-qPCR was performed to check the expression of *A. pisum* in PNA_BucCls -and PNA_ mm-treated aphid nymphs. The RT-qPCR protocol has been previously described. The KCISTOR_kaptinX1_F and KCISTOR_kaptinX1_R primers were used to amplify LOC100158964 (*kptn*/kaptin) gene, while the LOC107882452 (RT_Apisum) gene was amplified by RT_Apisum_1F and RT_Apisum_1R. The rpL7_F and rpL7_R primers were used to amplify the *A. pisum rpL7* gene, which served as a normalization ref.^53^ Log-transformation was applied to the *A. pisum* gene expression for subsequent statistical analysis.

#### Aphid survival analysis

The likelihood of aphid nymphs surviving for a prolonged period after PNAs treatment is tested in this study. A survival analysis was performed to determine whether there is a significant difference in survival probability between second instar aphid nymphs treated with 10 μM PNA_BucCls, PNA_mm or group treated with 12 mM CaCl_2_ solution (Group CaCl2). Following the injection, the treated aphid nymphs were housed in plastic cases, with eight aphids per container, each containing a broad bean seedling. The survival of aphids was quantified daily for 7 days.

### Quantification and statistical analysis

All data analyses were performed using the R (Ver 4.2.2) and relevant packages, including "devtools" (Ver. 2.4.5), "DescTools" (Ver. 0.99.50), and "dplyr" (Ver. 1.1.4).^42^^,^^43^^,^^44^^,^^45^ For data visualization, boxplots were generated using the "ggpubr" (Ver. 0.6.0) while raincloud plots were prepared by using "smplot2" (Ver. 0.1.0) packages in R.^46^^,^^47^

Survival analysis was carried out using various packages such as "survival" (Ver. 3.5-7) and "survminer" (Ver. 0.5.0).^48^^,^^49^ Kaplan-Meier curves were plotted using the "ggplot2" (Ver. 3.4.4) and "ggsurvfit" (Ver. 1.0.0) packages.^50^^,^^51^

Data were first evaluated for normality and homoscedasticity using the Shapiro-Wilk and Levene's tests, respectively. Based on these results, appropriate statistical tests were selected. RT-qPCR and qPCR data were analyzed using one-way ANOVA either with Fisher’s LSD or Dunnett’s post hoc tests. Kruskal-Wallis test followed by pairwise Wilcoxon rank sum post hoc test was applied to non-parametric data. Aphid fecundity and parturition timing were evaluated using one-way ANOVA with Tukey’s HSD post hoc test. Survival data were analyzed by log-rank test. Two-group comparisons in the supplementary figures were analyzed using one-tailed Student’s *t*-tests.

All statistical analyses were conducted with a significance level of α=0.05. Asterisks indicate statistical significance: *p* < 0.05 (∗); *p* < 0.01 (∗∗); *p* < 0.001 (∗∗∗); NS, not significant. Statistical details, including test types, *p* values, sample size (*n*) are also reported in the results and figure legends. In all cases, *n* refers to the number of individual aphid nymphs. Data are presented as mean±*SD* or median±*SD*, as specified.
