# Supplementary material for: Targeted disruption of the cls gene in Buchnera aphidicola impairs membrane integrity and host symbiont dynamics
Source: iScience. 2025 Jul 22;28(8):113178. doi: 10.1016/j.isci.2025.113178 (PMC12356351; doi:10.1016/j.isci.2025.113178)
Supplement: Document S1. Figures S1–S5 and Tables S1–S6 [file mmc1.pdf]

iScience, Volume *28*

Supplemental information

Targeted disruption of the *cls* gene in *Buchnera aphidicola* impairs membrane integrity and host symbiont dynamics

Kathrine Xin Yee Tan and Shuji Shigenobu

Figure S1

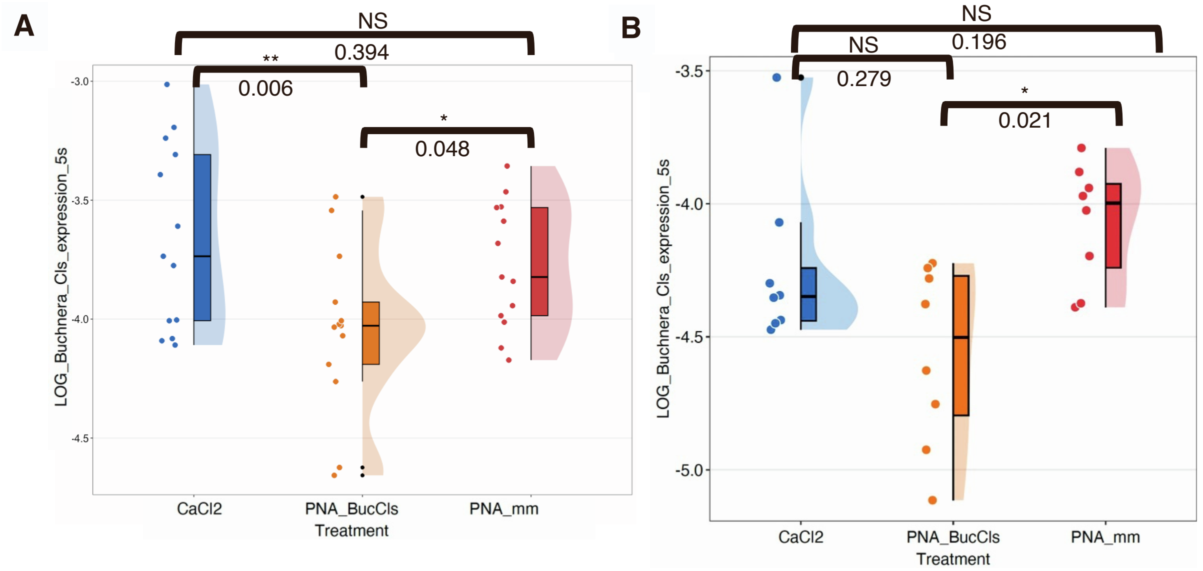

Figure S1: *Buchnera cls* gene expression was significantly reduced in 24 and 42 h after treated with anti-*cls* PNAs, Related to Figure 2 (A) *Buchnera cls* gene expression showed a marked reduction in aphid nymphs treated with PNA_BucCls compared to those treated with PNA_mm and CaCl2. *cls* gene expression was normalized by 5S rRNA gene. Targeted *Buchnera* gene expressions were quantified using RT-qPCR 24 h after PNA injection. Second instar aphid nymphs were injected with 10 μM peptide-conjugated anti-*cls* PNAs (PNA_BucCls) or control PNAs (CaCl2 = -3.66±0.391 (*M*±*SD*), PNA_BucCls = -4.05±0.348, PNA_mm = -3.77±0.266, *n* =13; ANOVA, $F(2, 36) = 4.46$, $p = 0.019$) (post hoc: Fisher's LSD test) (B) *Buchnera cls* gene expression was significantly reduced in aphid nymphs in PNA_BucCls than PNA_mm and CaCl2 groups (CaCl2 = -4.35±0.317 (*Mdn*±*SD*), PNA_BucCls= -4.50± 0.340, PNA_mm =-4.00±0.225, *n* = 8; Kruskal-Wallis test, $H(2) = 7.28$, $p = 0.026$) (post hoc: pairwise Wilcoxon rank sum test). Targeted *Buchnera* gene expressions were quantified using RT-qPCR 42 h after PNAs injection. Second instar aphid nymphs were injected with 15 μM peptide-conjugated anti-*cls* PNAs. Each dot indicates a single treated aphid nymph.

Figure S2

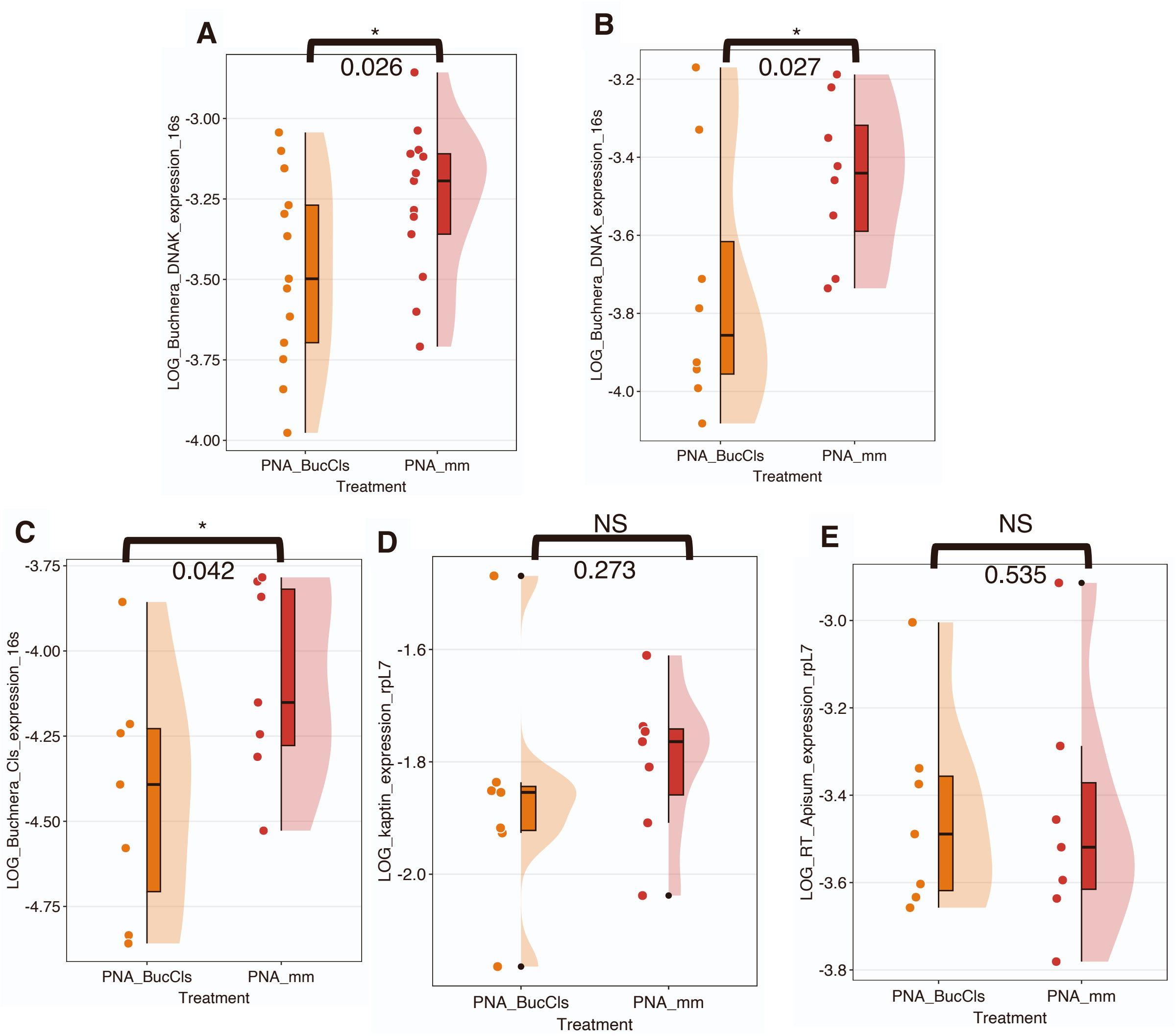
A
*
0.026
LOG_Buchnera_DNAK_expression_16s
-3.00
-3.25
-3.50
-3.75
-4.00
PNA_BucCls
PNA_mm
Treatment
B
*
0.027
LOG_Buchnera_DNAK_expression_16s
-3.2
-3.4
-3.6
-3.8
-4.0
PNA_BucCls
PNA_mm
Treatment
C
*
0.042
LOG_Buchnera_Cls_expression_16s
-3.75
-4.00
-4.25
-4.50
-4.75
PNA_BucCls
PNA_mm
Treatment
D
NS
0.273
LOG_kaptin_expression_rpL7
-1.6
-1.8
-2.0
PNA_BucCls
PNA_mm
Treatment
E
NS
0.535
LOG_RT_Apisum_expression_rpL7
-3.0
-3.2
-3.4
-3.6
-3.8
PNA_BucCls
PNA_mm
Treatment

Figure S2: The expression of untargeted *Buchnera* and *A. pisum* genes under peptide conjugated anti-*cls* PNAs, Related to Figure 2 (A) *Buchnera dnaK* gene expression in aphid nymphs treated with PNA_BucCls was significantly lower than that in those treated with PNA_mm in 24 h. *dnaK* gene expression was normalized by 16S rRNA gene expression. Second instar aphid nymphs were injected with 10 µM peptide-conjugated anti-*cls* PNAs or control PNAs. (PNA_BucCls = -3.47±0.295 (*M*±*SD*), PNA_mm = -3.26±0.238, n = 13; t (24) = -2.05, p = 0.026). (B) *Buchnera dnaK* gene expression in aphid nymphs treated with PNA_BucCls was significantly lower than those treated with PNA_mm in 42 h. Second instar aphid nymphs were injected with 15 µM peptide-conjugated anti-*cls* or control PNAs. (PNA_BucCls = -3.74±0.328 (*M*±*SD*), PNA_mm = -3.45±0.204, n = 8; t (14) = -2.11, p = 0.027). (C) *Buchnera cls* gene expression in aphid nymphs treated with PNA_BucCls was significantly lower than those treated with PNA_mm in 24 h (PNA_BucCls = -4.43±0.361 (*M*±*SD*), PNA_mm = -4.09±0.291, n = 7; t (12) = -1.89, p = 0.042). (D) The analysis of *A. pisum kaptin* (*kptn*/LOC100158964) gene expression, performed using the same RNA samples, revealed no significant difference between aphids treated with PNA_BucCls and those treated with PNA_mm. Expression levels were normalized to *A. pisum rpL7* (PNA_BucCls = -1.86± 0.206 (*M*±*SD*), PNA_mm = -1.80±0.137, n = 7; t (12) = -0.62, p = 0.273). (E) Similarly, the expression of LOC107882452 (*RT_Apisum*) showed no significant variation between the PNA_BucCls-treated group and the PNA_mm-treated group. (PNA_BucCls = -3.44±0.230 (*M*±*SD*), PNA_mm = -3.46±0.284, n = 7; t (12) = 0.090, p = 0.535). Analyses were conducted using one-tailed Student's t-test.

Figure S3

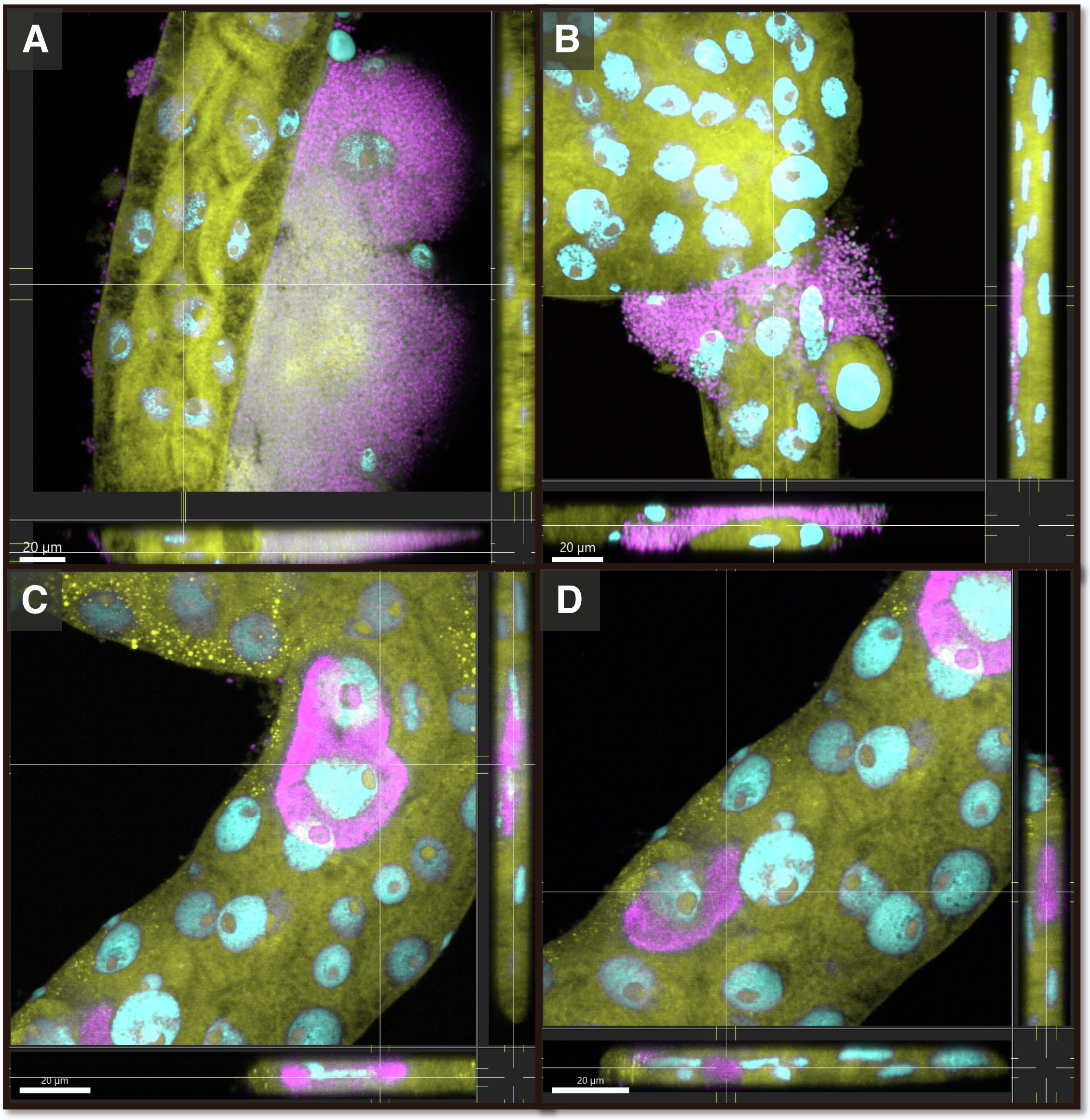

Figure S3: Localization of *Buchnera* in aphid guts following PNAs treatment, highlighting bacteriocyte overlap or gut invasion, Related to Figures 5-6. This figure illustrates two scenarios: bacteriocytes merely overlapping with the aphid gut or *Buchnera* cells seemingly within the gut. Aphid nymphs were injected with either PNA_BucCls (A) or PNA_mm (B) (15 μM in 12 mM $CaCl_2$ solution) and dissected at different time points (PNA_BucCls: 68 h; PNA_mm: 44 h). Cy5-conjugated ApisP2a was used to visualize *Buchnera* localization. (A) In PNA_BucCls-treated aphids, no *Buchnera* FISH probe signal was observed in the gut, and orthogonal views of z-stack confocal images confirmed the absence of *Buchnera* signals (z dimension = 0.35 μm per slice, 62 slices). (B) PNA_mm-treated aphids with no detectable *Buchnera* FISH probe signals in the gut (z dimension = 0.35 μm per slice, 78 slices). (C-D) Further detection of *Buchnera* FISH probe signals in the gut of *A. pisum* at 44 h post-injection. Orthogonal views of z-stack confocal images confirmed the presence of *Buchnera* signals two distinct locations within a single gut (z dimension = 0.22 μm per slice, 61 slices). For all observations, dissected guts were stained with DAPI to mark nuclei (cyan), *Buchnera*-specific FISH probe signals appeared magenta, and actin filaments were visualized with phalloidin peptide (yellow). (Scale bars: A-D = 20 μm)

Figure S4

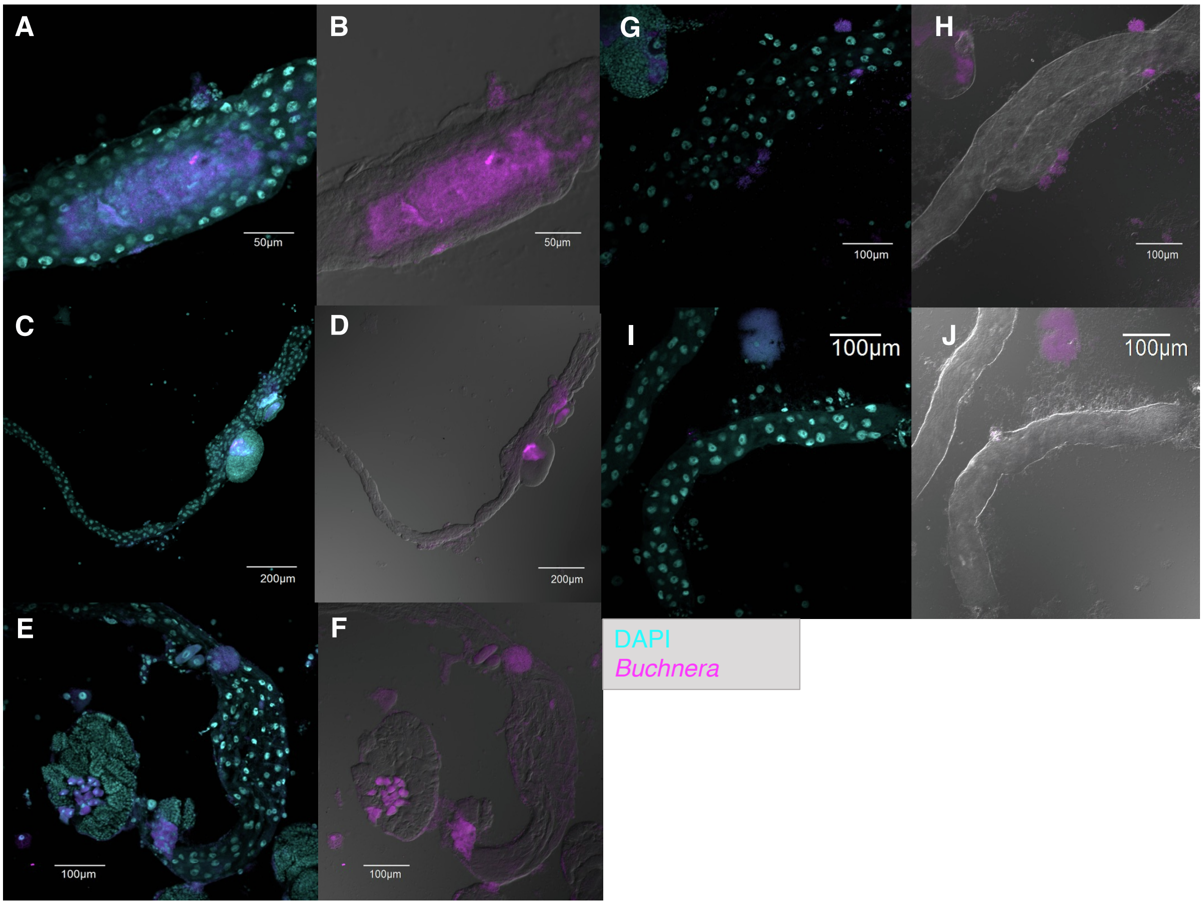

Figure S4: Detection of *Buchnera* cells in *A.pisum* gut of PNAs and negative control treatments, Related to Figure 5. *Buchnera* signals were detected only in the gut of aphid treated with PNA_BucCls but not the other treatments. (A-B) Gut of *A. pisum* injected with PNA_BucCls (15 µM in 12 mM $CaCl_2$ solution) and dissected 44 h post injection. (C-D) Gut of *A. pisum* treated 15 µM PNA_mm 44 h after treatment. (E-F) Gut of *A. pisum* treated 12mM $CaCl_2$ 44 h after treatment. (G-H) Gut of *A. pisum* treated 15 µM PNA_GroEL 48 h after treatment. (I-J) Gut of *A. pisum* treated 15 µM PNA_GroEL 68 h after treatment. *Buchnera* specific FISH probe revealed the localization of *Buchnera* in aphid gut (magenta). Nuclei of cells were marked with the DAPI solution (cyan). All images were taken using a 20 X objective lens except C-D where images were taken using 10 X objective lens. (Scale bars: A-B = 50 µm; C-D = 200 µm; E-J = 100 µm)

Figure S5

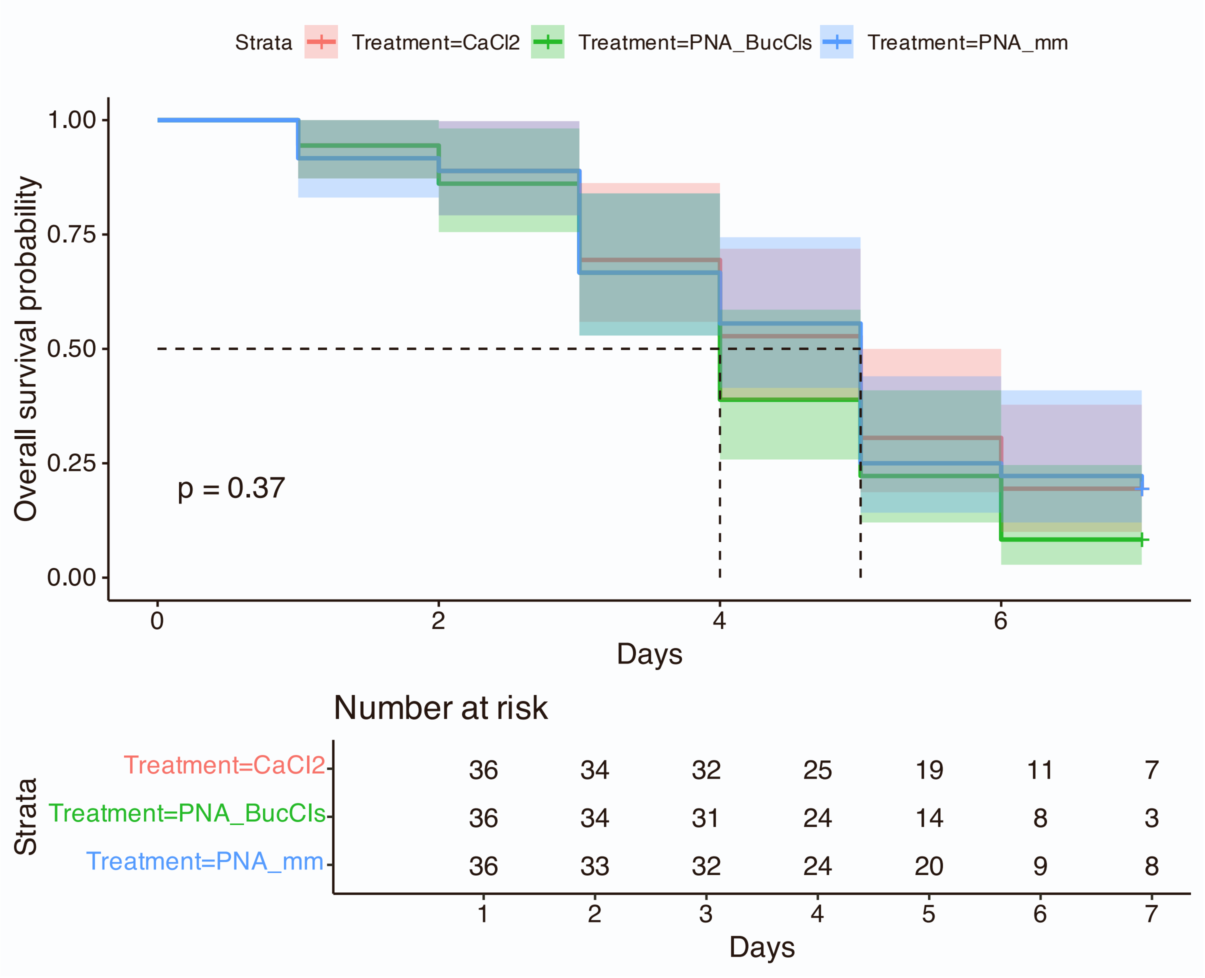

Figure S5: Kaplan-Meier survival curves of aphid nymphs treated with $CaCl_2$, PNA_BucCls and PNA_mm. Second instar aphid nymphs were injected with 10 µM peptide-conjugated antisense *cls* PNA (PNA_BucCls; green plot) or control PNAs (PNA_mm; blue plot), 36 aphid nymphs for each treatment. Additional negative control group, CaCl2 included 36 aphid nymphs treated with 12 mM $CaCl_2$ solution (red plot). There is no significant difference in survival probability between the three treatments (p = 0.37 > 0.05, log-rank test).

Table S1: Reverse complement sequence matches of anti-*cls* PNAs in *Buchnera aphidicola* str. APS genome, Related to Figure 1

Gene Name	CDS start position	CDS end position	Strand	Query start position	Query end position	Query strand	Comment	Extra Comment	Query sequence
cds-WP_010896038.1 (*cls*/ BucCls)[a]	298238	299698	- NC_002528.1	299694	299703	1	reverse complement match	Translational Start Site Included	TCCATCCATT

[a.](gene name/ primer name)

Table S2: *Buchnera cls* gene targeting PNAs, Related to Figure 1

PNA designation	PNA sequence	*Tm* (at 4 μM; °C)	Targeting gene	Target site	Off-target gene
PNA_mm	$(RXR)_4XB^a$-O-gcgatttgtc-NH_2	55.8	-	-	-
PNA_BucCls	$(RXR)_4XB$-O-tccatccatt-NH_2	48.6	*cls*	-5 to +5	-

[a.] In the cell-penetrating peptide conjugate, $(RXR)_4XB$ = RXRRXRRXRRXRXB, X stands for 6-aminohexanoic acid, B stands for beta-alanine while R stands for arginine.

Table S3: Statistical analysis of *Buchnera cls*, *groEL*, *murA*, *murC*, *flgC* and *ftsZ* genes' expression under different PNAs and control treatments, Related to Figure 7

Gene	Treatment	n	*M±SD*	ANOVA *F*(df1, df2)	*F*	*p*	Dunnett comparison	Mean Difference	Lower CI	Upper CI	*p*.adjust	
BuchCls (*cls*)	CaCl2	7	-3.92±0.357	*F*(3, 21)	0.772	0.001	CaCl2-PNA_mm	0.105	-0.319	0.528	0.865	
	PNA_BucCls	6	-4.57±0.230			**	PNA_BucCls-PNA_mm	-0.543	-0.983	-0.104	0.014	*
	PNA_GroEL	6	-4.58±0.360				PNA_GroEL-PNA_mm	-0.553	-0.993	-0.114	0.012	*
	PNA_mm	6	-4.03±0.212									
BuchGroEL (*groEL*)	CaCl2	7	-1.69±0.203	*F*(3, 21)	4.715	0.011	CaCl2-PNA_mm	-0.053	-0.532	0.426	0.985	
	PNA_BucCls	6	-2.15±0.430			*	PNA_BucCls-PNA_mm	-0.515	-1.013	-0.018	0.041	*
	PNA_GroEL	6	-2.20±0.473				PNA_GroEL-PNA_mm	-0.563	-1.060	-0.066	0.025	*
	PNA_mm	6	-1.64±0.171									
BuchMurA (*murA*)	CaCl2	7	-3.51±0.340	*F*(3, 21)	5.990	0.004	CaCl2-PNA_mm	0.051	-0.487	0.590	0.990	
	PNA_BucCls	6	-4.31±0.413			**	PNA_BucCls-PNA_mm	-0.756	-1.315	-0.197	0.007	**

	PNA_GroEL	6	-3.93±0.456				PNA_GroEL-PNA_mm	-0.374	-0.933	0.185	0.241	
	PNA_mm	6	-3.56±0.314									
BuchMurC (*murC*)	CaCl2	7	-3.78±0.302	*F*(3, 21)	3.952	0.022	CaCl2-PNA_mm	0.085	-0.506	0.677	0.967	
	PNA_BucCls	6	-4.53±0.517			*	PNA_BucCls-PNA_mm	-0.657	-1.271	-0.043	0.035	*
	PNA_GroEL	6	-4.15±0.450				PNA_GroEL-PNA_mm	-0.279	-0.893	0.335	0.531	
	PNA_mm	6	-3.87±0.405									
BuchFlgC (*flgC*)	CaCl2	7	-3.30±0.662	*F*(3, 20)	7.128	0.002	CaCl2-PNA_mm	-0.192	-0.947	0.563	0.855	
	PNA_BucCls	6	-3.94±0.374			**	PNA_BucCls-PNA_mm	-0.829	-1.613	-0.046	0.037	*
	PNA_GroEL	5	-4.42±0.614				PNA_GroEL-PNA_mm	-1.314	-2.136	-0.493	0.002	**
	PNA_mm	6	-3.11±0.416									
BuchFtsZ (*ftsZ*)	CaCl2	7	-3.21±0.242	*F*(3, 20)	5.523	0.006	CaCl2-PNA_mm	-0.024	-0.542	0.494	0.999	
	PNA_BucCls	6	-3.78±0.362			**	PNA_BucCls-PNA_mm	-0.587	-1.125	-0.049	0.031	*
	PNA_GroEL	5	-3.85±0.591				PNA_GroEL-PNA_mm	-0.662	-1.226	-0.099	0.020	*
	PNA_mm	6	-3.19±0.237									

Table S4: Statistical analysis of *Buchnera fliI* gene expression under different PNAs and control treatments using Kruskal-Wallis Test, Related to Figure 7

Gene	Treatment	n	*Mdn*±*SD*	Kruskal-Wallis χ^2(df)	*p*	Pairwise comparisons using Wilcoxon rank sum exact test	*p*.adjust(bonferroni)	
BuchFliI (*fliI*)	CaCl2	7	-3.95±0.453	$\chi^2(3) = 10.698$	0.013	PNA_BucCls-CaCl2	0.084	
	PNA_BucCls	6	-4.76±0.160		*	PNA_GroEL-CaCl2	0.308	
	PNA_GroEL	6	-4.67±0.419			PNA_mm-CaCl2	1.000	
	PNA_mm	6	-4.36±0.267			PNA_GroEL-PNA_BucCls	1.000	
						PNA_mm-PNA_BucCls	0.013	*
						PNA_mm-PNA_GroEL	1.000	

Table S5: Anti-*cls* PNAs reverse complement matches overlapping translational start sites in *Acyrthosiphon pisum* isolate AL4f genome

Gene Name	CDS start position	CDS end position	Strand	Query start position	Query end position	Query strand	Comment	Extra Comment	Query sequence
GeneID:107882452 (uncharacterized/ RT)[a]	32751370	32751756	- NC_042495.1	32751748	32751757	1	reverse complement match	Translational Start Site Included	TCCATCCATT
GeneID:115034171	102021822	102021942	- NC_042495.1	102021934	102021943	1	reverse complement match	Translational Start Site Included	TCCATCCATT
GeneID:100158964 (*kptn/* kaptin)	163540623	163540824	- NC_042494.1	163540816	163540825	1	reverse complement match	Translational Start Site Included	TCCATCCATT

GeneID:103310598	89916970	89922186	- NC_042494.1	89922178	89922187	1	reverse complement match	Translational Start Site Included	TCCATCCATT

[a.] (gene name/ primer name)

Table S6: List of primers used in qPCR and RT-qPCR analyses

Primer	Sequence	Primer length (bp)	Amplicon length (bp)	Target gene	Efficiency (%)
rpL7_F	GCGCGCCGAGGCTTAT	16	81	*A. pisum rpl7*	100.0
rpL7_R	CCGGATTTCTTTGCATTTCTTG	22			
BuchDnaK_F1018	GTTGGTGGTCAAACTAGAATGCCT	24	125	*dnaK*	101.8
BuchDnaK_R1142	ACTCCTCCCTGTACTGCAGC	20			
Buch_rrs_F274	AGGATAACCAGCCACACTGG	20	115	16S rRNA gene	98.0
Buch_rrs_R388	TCTTCATACACGCGGCATAG	20			
Buch_5S_F	CACCTGAATCCATTCCGAAC	20	92	5S rRNA gene	95.5
Buch_5S_R	TTCCTGGATTTGACCTACTCTCA	23			
BuchCls_2F	GGCAGATGATGTGGCAATAG	20	114	*cls*	128.0
BuchCls_2R	ATTTCAACCCAGGGGCTCT	19			
BuchGroEL_1_F	AAACTATCAGGCGGTGTTGC	20	226	*groEL*	
BuchGroEL_R	CACGCAAAGCAACTCGAATA	20			92.6

BuchMurA_F	ATGCAGGCTCAATTTGCTCT	20	242	*murA*	172.5
BuchMurA_R	ATACCTGCCGCAATACATCC	20			
BuchMurC_F	ACGGGCATCATCCTACAGAA	20	186	*murC*	96.8
BuchMurC_R	CATTTGCAGAGTAGACATGCAG	22			
BuchFliI_F	GCTATGCTATGGCACAACGA	20	205	*fliI*	105.1
BuchFliI_R	CGAGCAAGATGTGAAACTGG	20			
BuchFlgC_F	TCGCGAAACAAGTCGTATTTT	21	215	*flgC*	161.7
BuchFlgC_R	AGCCTGGTAGCTTCTTGCTG	20			
BuchFtsZ_F	GGATATGCGATGATGGGAAC	20	219	*ftsZ*	139.4
BuchFtsZ_R	AACCACTGTTGCGTGATCTG	20			
KCISTOR_kaptinX1_F	CGGCGACCAAAAGATTGTTA	20	187	*A. pisum kptn/* kaptin (LOC100158964)	105.5
KCISTOR_kaptinX1_R	CCCGAAAACGAAATCATCAA	20			
RT_Apisum_1F	GGGGAAGAGGAACAAAAAGTG	21	150	*A. pisum* (LOC107882452)	141.4
RT_Apisum_1R	CACCGTTATGGTTTCGGTTT	20			
